# HPLC Analysis and In Vitro and In Silico Evaluation of the Biological Activity of Polyphenolic Components Separated with Solvents of Various Polarities from *Helichrysum italicum*

**DOI:** 10.3390/molecules28176198

**Published:** 2023-08-23

**Authors:** Dimitar Bojilov, Stanimir Manolov, Sezan Ahmed, Soleya Dagnon, Iliyan Ivanov, Gabriel Marc, Smaranda Oniga, Ovidiu Oniga, Paraskev Nedialkov, Silviya Mollova

**Affiliations:** 1 Department of Organic Chemistry, Faculty of Chemistry, University of Plovdiv, 24 “Tsar Assen” Street, 4000 Plovdiv, Bulgaria; nsahmed1998@gmail.com (S.A.); solbono@abv.bg (S.D.); iiiliyan@abv.bg (I.I.); 2 Department of Pharmaceutical Chemistry, Faculty of Pharmacy, “Iuliu Hațieganu” University of Medicine and Pharmacy, 41 Victor Babeș Street, RO-400010 Cluj-Napoca, Romania; marc.gabriel@umfcluj.ro (G.M.); ooniga@umfcluj.ro (O.O.); 3 Department of Therapeutic Chemistry, “Iuliu Hațieganu” University of Medicine and Pharmacy, 12 Ion Creangă Street, RO-400010 Cluj-Napoca, Romania; smaranda.oniga@umfcluj.ro; 4 Department of Pharmacognosy, Faculty of Pharmacy, Medical University of Sofia, 2 Dunav Street, 1000 Sofia, Bulgaria; pnedialkov@pharmac.mu-sofia.bg; 5 Institute of Roses, Essential and Medical Plants, Agricultural Academy, 49 Osvobozhdenie Blvd., 6100 Kazanlak, Bulgaria; sysi_a@abv.bg

**Keywords:** *Helichrysum italicum*, HPSA, HRSA, MChA, NOSA, IAD, ATA, *in silico* molecular docking and dynamics, polyphenols, arzanol, bitalin A

## Abstract

*Helichrysum italicum* has piqued the interest of many researchers in recent years, mostly for its essential oil, but increasingly for its polyphenolic content as well. In the current study, we examine the polyphenolic composition of *H. italicum* grown in Bulgaria. The polyphenolic complex was fractionated with solvents of various polarities, including hexane, chloroform, ethyl acetate, and butanol, in order to assess the biological impact of the components. HPLC-PDA and UHPLC-MS/MS were used to examine all fractions. The green coffee fingerprint profile was employed as a “surrogate standard” in the polyphenolic components detection approach. From the UHPLC-MS/MS analysis, we identified 60 components of the polyphenolic complex such as quercetin 3-*O*-glucuronide, quercetin acetyl-glycoside, isorhamnetin acetyl-glycoside, isorhamnetin caffeoyl-glycoside, quercetin caffeoyl-malonyl-glycoside, isorhamnetin coumaroyl-glycoside, coumaroyl-caffeoylquinic acid, and diCQA-acetyl-derivative were first reported in the composition of *H. italicum*. The biological activity of the fractions was evaluated *in vitro* and *in silico*, which included the fight against oxidative stress (hydrogen peroxide scavenging activity (HPSA), hydroxyl radical scavenging activity (HRSA), metal-chelating activity (MChA)) and nitrosative (nitric oxide scavenging activity) (NOSA)), *in vitro* anti-inflammatory, and anti-arthritic activity. Results are presented as IC_50_ ± SD μg/mL. The analysis showed that the EtOAc fraction was characterized by highest HPSA (57.12 ± 1.14 μg/mL), HRSA (92.23 ± 1.10 μg/mL), MChA (5.60 ± 0.17 μg/mL), and NOSA (89.81 ± 2.09 μg/mL), while the hexane and chloroform fractions showed significantly higher *in vitro* anti-inflammatory activity (30.48 ± 2.33 μg/mL, 62.50 ± 1.69 μg/mL) compared to the standard ibuprofen. All three fractions showed potential anti-arthritic activity (102.93 ± 8.62 μg/mL, 108.92 ± 4.42 μg/mL, 84.19 ± 3.89 μg/mL).

## 1. Introduction

The traditions of folk medicine in many countries around the world have preserved the millennia-old knowledge of the beneficial effects of herbs on health. Herbs, often known as medicinal or medical plants and medicinal herbs, are a vast category of plants used in medical and veterinary practice for illness prevention and treatment. They have been used by all cultures and ethnic groups throughout history to improve human health. They are the oldest type of pain and disease therapy known to humankind, but they are also a significant source of contemporary medicine.

In the 3rd–2nd century BC, the book *Historia Plantarum* by the Greek Theophrastus of Eresos mentions the species *Helichrysum* for the first time for medicinal uses [1]. The genus *Helichrysum* consists of about 600 species of plants [2]. *H. italicum* (*Roth*) *G. Don* belongs to the *Asteraceae* family. Known as curry or the everlasting plant, its name is derived from the Greek words *helios* (sun) and *chrysos* (gold) [3].

One of the most studied representatives is *H. italicum*. This herb is also known to be called “the herb of the Mediterranean” because it is endemic to the Mediterranean region [4].

The chemical composition of *Helichrysum italicum* contains a large and diverse group of secondary metabolites. It has been reported in the literature that various extracts of the plant have shown remarkable biological properties [4,5,6,7,8]. Besides being rich in flavonoids, the plant is characterized by an exceptional accumulation of pyrones, phloroglucinols, acetophenones, and tremetones. It is found in several taxonomic groupings across the plant world and has various pharmacological qualities such as antioxidant and anti-inflammatory actions [4,7,8,9].

Different types of extracts obtained from *H. italicum* were studied by Sala et al. [6], and they reported the presence of acetophenone derivatives: 4-hydroxy-3-(3-methyl-2-butenyl) acetophenone, 4-hydroxy-3-(2-hydroxy-3-isopentenyl) acetophenone, and 1-[2,3-dihydro-2-[1-(Hydroxymethyl)ethenyl]-5-benzofuranyl]-ethanone (bitalin A) [9,10,11,12], (3-(3-methyl-2-butenyl) acetophenone-4-*O*-*β*-glucopyranoside) and (12-hydroxytremetone-12-*O*-*β*-D-glucopyranoside), 3-(2-hydroxyethyl) acetophenone-4-*O*-*β*-D-glucopyranoside, and maltol-*β*-D-glucopyranoside [4].

Also identified are a series of tremetones, acetoxytremetone 10-hydroxytremetone, 3-acetoxy-10-hydroxytremetone, and the phloroglucinols arzanol, methylarzanol, and the pyrones helipyrone, and micropyrone [4,5,6,7].

From the group of caffeoylquinic acids, 3-caffeoylquinic acid (neochlorogenic acid), 5-caffeoylquinic acid (chlorogenic acid), 4-caffeoylquinic acid (cryptochlorogenic acid), 5-*O*-caffeoyl-4-methylquinic acid, 1,3-dicaffeoylquinic acid, 4,5-dicaffeoylquinic acid, 3,4-dicaffeoylquinic acid, 1,5-dicaffeoylquinic acid, 1,4-dicaffeoylquinic acid, 3,5-dicaffeoylquinic acid methyl ester, have been identified [11,13]. Previous studies have reported other phenolic acids such as *p*-coumaric acid, caffeic acid, ferulic acid, gallic acid [12,14], and caffeoyltartaric acid (chicoric acid) [15].

Of the flavonoid glycosides, quercetin 3-*O*-glucoside, kaempferol 3-*O*-glucoside, isorhamnetin 3-*O*-glucoside, quercetin 3’-*O*-glucoside, tiliroside, and of the aglycones gnaphalin were identified [11]. The composition also includes isoquercetin, [13], luteolin-7-glucoside, isoquercetrin, apigenin-7-glucoside, hyperoside, quercetin, 4,2′,4′,6′-tetrahydroxychalcone-2′-glucoside, naringenin-4-glucoside, [16,17], naringenin-glycoside, [18], naringenin-7-*O*-glucoside, naringenin-diglycoside, narirutin, naringenin-4-glucoside-7-rutinoside, hesperidin, kaempferol-rutinoside, myricetin-glucoside, [14], and helichrysin [13].

A series of flavonoid aglycones have also been identified: galangin, galangin-3-methyl ether, 8-hydroxygalangin, 8-hydroxygalangin-3-methyl ether, gnaphalin, kaempferol-3-methylether, herbacetin-3-methylether, quercetin-3-methylether, gossypetin-3-methylether, gossypetin-3,8-dimethylether, pinocembrin, apigenin, luteolin, naringenin, methoxyluteolin, kaempferol epigallocatechin-3-*O*-gallate [14,19,20].

*H. italicum* extracts have been shown to have antibacterial, antiviral, antioxidant, and anti-inflammatory action and to successfully inhibit the enzymes acetylcholinesterase, tyrosinase, and α-glucosidase.

Ether, methanol, and ethanol extracts of *H. italicum* have shown high antibacterial activity against different strains *Staphylococcus aureus*, *Streptococcus mutans*, *Micrococcus luteus*, *S. aureus*, *Compylobacter coli*, *E. coli*, *Salmonella infantis*, *Bacillus cereus*, and *Listeria monocytogenes*, antifungal activity against *Pythium ultimum*, and bactericidal activity against *S. epidermidis* [1,8,9,21,22,23,24,25,26,27,28,29,30,31]. *H. italicum* extracts also have antiviral activity against herpes simplex virus type 1 (HSV-1) [31], which is believed to be due to the flavonoids apigenin and luteolin present in *H. italicum* extracts, which has been proven in other studies [32,33]. Good anti-HIV activity was shown by the phloroglucinol arzanol isolated from *H. italicum subsp. microphyllum*, suggesting that it inhibits HIV-1 replication in T-lymphocytes.

A number of authors have reported the antioxidant activity of different *H. italicum* extracts [21,29,34,35,36,37,38,39], using different methods for evaluation, including FRAP, DPPH, ABTS, and CUPRAC.

Interesting results for anti-inflammatory activity of methanol extracts of *H. italicum* were obtained when testing their ability to inhibit arachidonic acid metabolism by two different *in vitro* methods [8]. The flavonoids gnaphalin and pinosembrin were found to inhibit leukotriene B4 production, and 4-hydroxy-3-(3-methyl-2-butenyl) acetophenone inhibited cyclooxygenase-1 (COX-1) activity [7,21]. Other authors found that the anti-inflammatory activity of the methanolic extract has a mechanism of action similar to that of corticosteroids [4,7,8].

For the first time, Goncalves and co-authors reported that the methanol extract of *H. italicum* inhibited the enzymes acetylcholinesterase AChE, tyrosinase, and *α*-glucosidase. It is the same extract that shows a high inhibitory potential against AChE (78.29%), tyrosinase (74.13%), and *α*-glucosidase (96.65%), enzymes associated with Alzheimer’s disease, which is due to the high content of caffeoylquinic and dicaffeoylquinic acids in the extract [21,40].

*Helichrysum italicum* has been cultivated in Bulgaria in recent years, although its polyphenol content has not yet been studied. As a consequence, the purpose of this study was to look into the ability of *H. italicum*’s polyphenolic composition in terms of avoiding damaging radicals caused by oxidative and nitrosative stress, metal-chelating activity, and *in vitro* anti-inflammatory and anti-arthritic activity. Many crucial steps must be taken to attain the aim, such as fractionation of the polyphenolic content with various polarity solvents. Polyphenols may be separated into polar, slightly polar, and nonpolar polyphenolic groups using this procedure. The second stage is to analyze individual polyphenol groups using HPLC-DAD and UHPLC-MS. This is linked to the identification, quantification, and mass spectral analysis of the polyphenolic complex. The third goal is to investigate the structure–activity connection of distinct polyphenol groups.

## 2. Results and Discussion

### 2.1. Fractionation of the Polyphenolic Complex with Solvents of Different Polarity—Yield

Fractionation of polyphenolic composition is an approach that solves two important tasks. The first task is to eliminate the influence of the matrix and thus facilitate the analysis and identification of the components by UHPLC-MS/MS. The second task in studying biological activity is to assess which classes of polyphenolic components exhibit greater activity. This allows us to establish a link between structure and activity. Through fractionation, the components of the polyphenolic complex are separated into fractions of different polarity. Therefore, we used solvents with varying polarities, such as hexane, chloroform, ethyl acetate, and butanol, in the separation of the polyphenolic complex [41].

**Figure 1:**
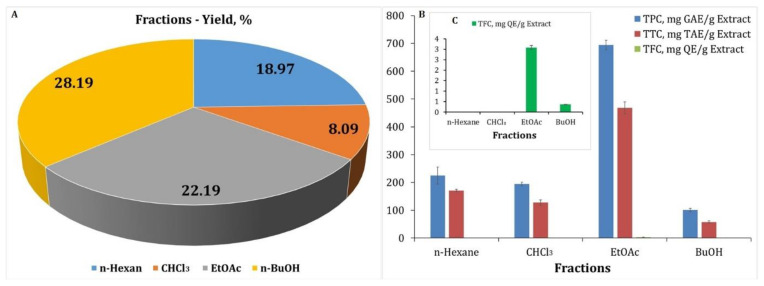
Yields (%) of the hexane, chloroform, ethyl acetate, and butanol fractions. (**A**) Total phenolic content (TPC), total tannin content (TTC), and total flavonoid content (TFC) of different fractions obtained from *H. italicum*. TPC and TTC results are presented as mg GAE/g Extr and mg TAE/g Extr, respectively (**B**). TFC results are presented as mg QE/g Extr (**C**).

After fractionation of the polyphenol complex, we calculated the yields of the hexane, chloroform, ethyl acetate, and butanol fractions (Figure 1A). The butanol fraction had the highest yield (28.19%), followed by the ethyl acetate fraction (22.19%), and the lowest yield was the chloroform fraction (8.09%) (Table 1, Figure 1A).

### 2.2. Determination of the Content of Total Phenols (TPC), Tannins (TTC) and Flavonoids (TFC)

Polyphenols found in medicinal plants are important for human health because they contribute considerably to antioxidant activity, anti-inflammatory activity, anti-arthritic activity, UV-B protection, and other activities. As a result, when studying medicinal plants, it is critical to assess the total phenolic content (TPC), total tannin content (TTC), and total flavonoid content (TFC) in various extracts, fractions, and tinctures. After the fractionation of the polyphenolic complex, we determined TPC, TTC, and TFC in the different fractions of *H. italicum* (Table 1 and Figure 1B,C).

The butanol fraction has the largest yield but the lowest concentration of phenols, tannins, and flavonoids. In the ethyl acetate fraction, the content of phenols, tannins, and flavonoids is the highest. The presence of flavonoids was not detected in the hexane or chloroform fractions. It is important to highlight that the procedure is spectrophotometric. The sensitivity of a method depends on the type of spectral examination. We discovered a number of flavonoids in more comprehensive analyses of the chloroform fraction using UHPLC-MS/MS, but their quantity was too low. UV spectra revealed that the components in the hexane and chloroform fractions absorb at λ_max_ = 280 nm, which is characteristic of phloroglucinols and tremetones. Their concentration was calculated using a gallic acid standard. The content of tremetones in the chloroform fraction was 26.84 mg/g Extr., while the amount of phloroglucinols in the hexane fraction was 7.98 mg/g Extr (Table 1). In the ethyl acetate fraction, the content of total flavonoids was 3.08 mgQE/g Extr, while in the butanol fraction it was 0.36 mgQE/g Extr (Table 1 and Figure 1C). TFC is a spectrophotometric and relative method. This method does not provide information on the type of flavonoids contained. It is in combination with it that chromatographic analysis is used. The results of the chromatographic analysis confirmed the low content of flavonoids. Using a HPLC-PDA method, we identified that quercetin flavonoids and their content in the ethyl acetate fraction was 2.24 mg/g Extr, and in the butanol fraction it was 1.56 mg/g Extr. From HPLC-PDA analysis, we found that the content of caffeoylquinic acids is the highest and reaches 45.82 mg/g Extr (Table 1). The activity of the fractions depends on the type and content of polyphenolic components.

### 2.3. Fingerprint Profile of Polyphenols of H. italicum Obtained by HPLC-PDA

One of the most powerful analytical approaches for quality control of medicinal herbs is the creation of a chromatographic fingerprint profile. This method changes the purpose of quality control from analysis of a single chemical component to analysis of the entire chemical composition in the herb [42].

**Figure 2:**
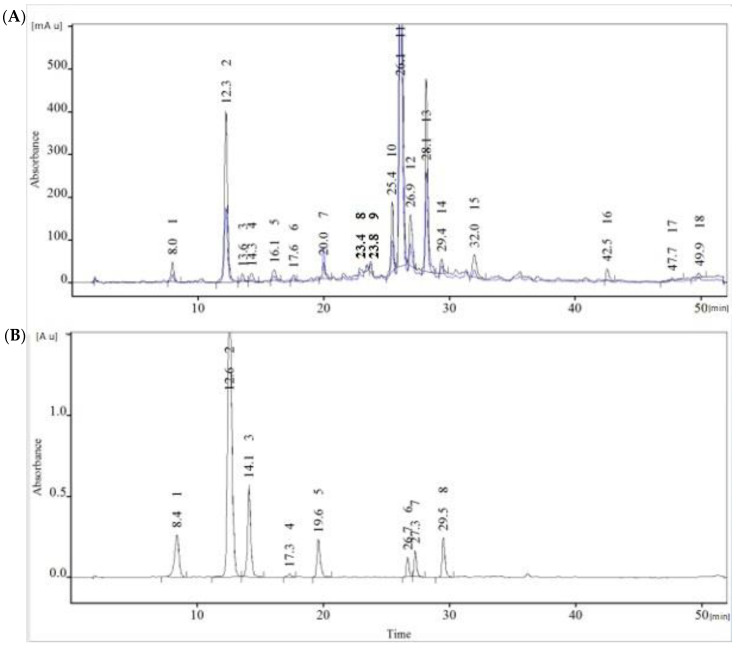
Analysis of polyphenols was done with HPLC-PDA (diode array detector). This detector enables polyphenols to be scanned at different wavelengths. In the present work, we used 329 nm (the blue line) for caffeoylquinic acids and 352 nm (the black line) for flavonoids. (**A**) Chromatographic profile of *H. italicum* polyphenol complex: peak 1—3-CQA (neochlorogenic acid), peak 2—5-CQA (chlorogenic acid), peak 3—4-CQA (cryptochlorogenic acid), peak 4—feruloylquinic acid, peak 7—quercetin glycoside, peak 11—3,4-di CQA, peak 12—3,5-di CQA, peak 13—4,5-di CQA. (**B**) Chromatographic profile of the polyphenolic complex of green coffee: peak 1*—*neochlorogenic acid, peak 2*—*chlorogenic acid, peak 3—4-CQA, peak 4*—*feruloylquinic acid, peak 5—feruloylquinic acid, peak 6—3,4-di CQA, peak 7—3,5-di CQA, peak 8—4,5-di CQA.

To obtain such a chromatographic profile, the ideal parameters for obtaining the greatest number of well-separated peaks must be chosen. For our study, we used a Kromasil C18 column, formic acid as an organic modifier, and acetonitrile. The gradient has several levels. We were able to separate the chlorogenic acid and dicaffeoylquinic acid isomers fairly successfully in this study (Figure 2A).

#### Polyphenolic Component Identification Using a Green Coffee “Surrogate Standard”

The identification of polyphenols is essential because in the study of biological activity, very often a connection is made between structure and activity. Different methods are applied to identify polyphenols, the most common being standards, by hydrolysis, by UV and MS/MS spectral data, and by using “surrogate standards.”

Analysis of the polyphenolic complex of green coffee extract, which we used as a surrogate standard, was performed on HPLC-PDA, under the same conditions used for *H. italicum*. As a result of this study, we obtained a chromatographic fingerprint profile of the green coffee polyphenol complex (Figure 2B). The polyphenolic complex of green coffee has been widely studied. Its components are identified and described by Clifford [43]. In Figure 2B, seven main peaks are clearly distinguished. Caffeoylquinic acids (mono- and di-) are the main components present in the polyphenolic complex of green coffee. By carefully controlling the method conditions, supported by UV spectra from the photodiode array detector and retention times, we can reliably identify the caffeoylquinic acids present in the chromatographic profile of *H. italicum*, using the “surrogate standard.”

In Figure 2B, peak 2 with retention time t_R_ = 12.6 min was identified as 5-*O*-caffeoylquinic acid (chlorogenic acid). In the profile of *H. italicum* (Figure 2A), analyzed under the same conditions, peak 2 with a retention time t_R_ = 12.3 min is clearly distinguished. UV data reveal a characteristic spectrum of chlorogenic acid with absorption at λ_max_ = 329 nm. Comparing the two profiles, the presence of the isomers of chlorogenic acid with t_R_ = 8.0 and t_R_ = 13.6 min is established. Our assumption that peaks 11, 12, and 13 of the *H. italicum* profile are 3,4-, 3,5-, and 4,5-dicaffeoylquinic acids, respectively, is further confirmed by the “surrogate standard” and by UHPLC-MS/MS analysis. In Figure 2, the retention times and spectral data match the two profiles.

After identifying the major components in the polyphenolic complex of *H. italicum*, its fractionation was performed. This method allows the components to be separated based on their polarity. The chromatographic examination reveals the distribution of polyphenolic components in the different extracts.

A chromatographic profile was prepared on the obtained fractions, and it was found that the chlorogenic acids are distributed between the polar solvents in the ethyl acetate and butanol fractions (Figure S1), while phloroglucinols, pyrones, and italipyrone are distributed in the hexane fraction (Figure S2) and tremetones and aglycones in the chloroform fraction (Figure S2). The results obtained by HPLC-PDA were confirmed by UHPLC-MS/MS. In the present work, we aim to investigate the biological activity of the individual fractions and determine the relationship between structure and activity. The content of flavonoids, chlorogenic acids, and non-flavonoid polyphenols in the fractions was determined against the standards quercetin, chlorogenic acid, and gallic acid. The amount of caffeoylquinic acids and quercetin flavonoids was highest in the ethyl acetate fraction and lowest in the butanol fraction (Table 1).

### 2.4. Identification of Polyphenolic Components through UHPLC-MS/MS

Combined chromatographic methods are commonly used in phytochemistry. They provide crucial information regarding the phytocomponent composition. The polyphenolic composition of *H. italicum* growing in Bulgaria is investigated in this study. The literature indicates that the polyphenolic content is quite variable. The HPLC-PDA approach offers information on the medicinal plant’s fingerprint profile. The major components are examined using this method. According to the HPLC-PDA study, the primary components are mono- and di-caffeoylquinic acids. Components that are in low amounts are difficult to identify. In order to achieve a comprehensive analysis of the polyphenolic components, it is necessary to use UHPLC-MS/MS. In this regard, polyphenols were fractionated with solvents of different polarity—hexane (n-H), chloroform (CHCl_3_), ethyl acetate (EtOAc), and butanol (n-BuOH). All fractions obtained were subjected to UHPLC-MS/MS analysis. For the identification of the components, we applied the two ionization modes (positive and negative mode). The results of the analysis confirm the data published in previous scientific studies with few exceptions (Table A1). The analysis proves the presence of tremetones, pyrones, italipyrone, phloroglucinols, flavonoids, and caffeoylquinic acids. Furthermore, by UHPLC-MS/MS analysis we identified quercetin 3-*O*-glucuronide, quercetin acetyl-glycoside, isorhamnetin acetyl-glycoside, isorhamnetin caffeoyl-glycoside, quercetin caffeoyl-malonyl-glycoside, isorhamnetin coumaroyl-glycoside, coumaroyl-caffeoylquinic acid, and diCQA-acetyl-derivative, which are first reported in the composition of *H. italicum*.

**Figure 3:**
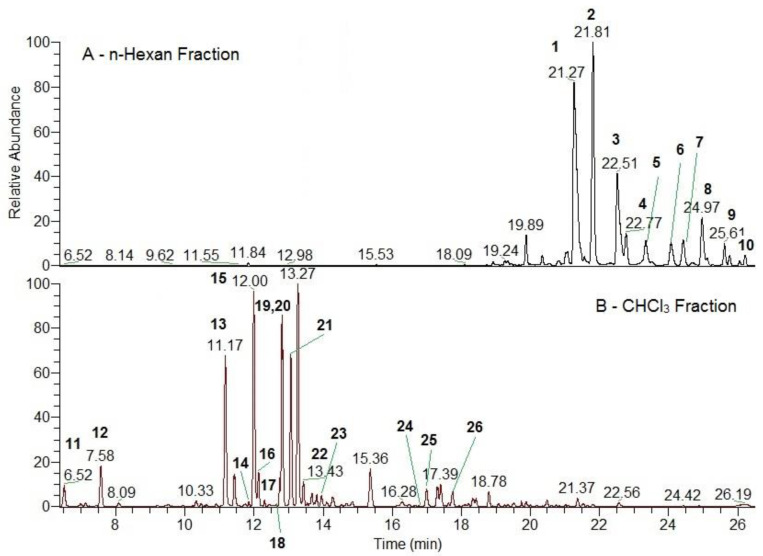
Total ion current (TIC) of phenolic compounds from *H. italicum* in n-Hexan fraction (**A**); total ion current (TIC) of phenolic compounds from *H. italicum* in CHCl_3_ fraction (**B**). Phloroglucinols (peaks—1–10) were identified in the n-Hexan fraction (**A**). Tremetones (peaks—12, 13, 17, 18, 22), flavonoids (14, 16, 19–26), caffeic acid (peaks 11), and italipyron-isomer (peaks 15) were identified in the CHCl_3_ fraction (**B**).

UHPLC-MS/MS analysis of the hexane fraction evidenced the presence of phloroglucinols (arzanol, methylarzanol, heliarzanol, and arzanol derivatives), italipyrones, and pyrones (Figure 3A, Table A1). Arzanols are the main components in the investigated fraction. From the literature, we understand that there are no data on a complete mass spectral study of the structure of non-flavonoid polyphenolic components such as phloroglucinols, tremetones, and italipyrones. This is an important step in MS identification of compounds because fragmentation produces ions that are characteristic of a class of compounds. It is in the present work under ESI(+) MS/MS conditions that we investigate the structure of phloroglucinols, tremetones, and italipyrones. Moreover, the positive mode gives better mass spectral information for these compounds.

**Scheme 1:**
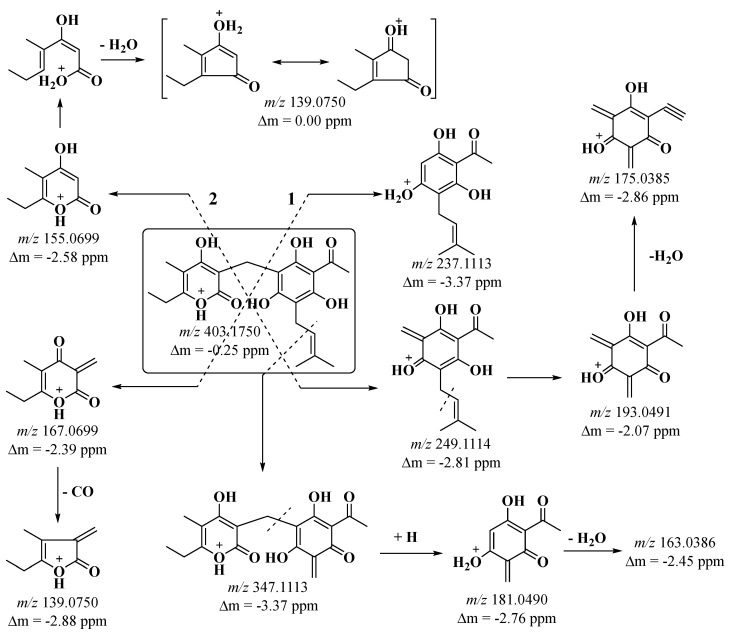
Proposed fragmentation of protonated arzanol [M+H]^+^.

The structure of arzanols contains a phloroglucinyl pyrone nucleus, i.e., the two structural fragments are connected by means of a methylene bridge (Scheme 1).

**Figure 4:**
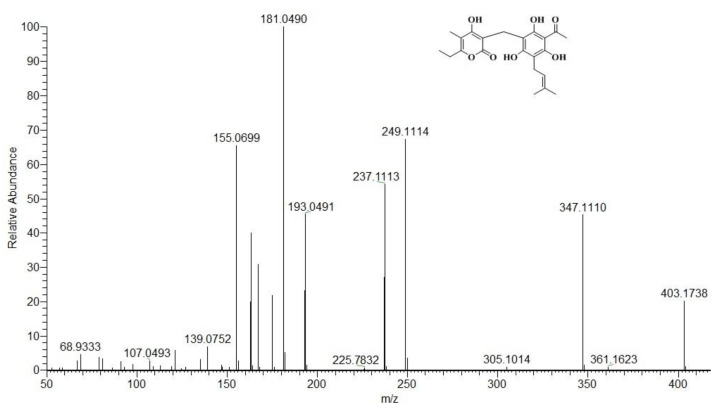
Mass spectrum of arzanol obtained by positive ion ESI-MS/MS.

Two cleavage pathways of the phloroglucinyl pyrone core are possible. Pathway 1 fragmentation produced the ions *m/z* 237 and *m/z* 167 (Scheme 1, Figure 4).

Ion *m/z* 167 is a precursor to ion *m/z* 139 with release of a neutral CO molecule. Path 2 leads to the production of ions *m/z* 249 and *m/z* 155 (Scheme 1, Figure 4). Ion *m/z* 249 undergoes loss of an isobutene fragment, yielding ion *m/z* 193. Ions *m/z* 193 and *m/z* 155 undergo rearrangement with release of a water molecule, generating the fragment ions *m/z* 175 and *m/z* 139. Arzanol with the C–C bond cleavage loses an isobutene fragment, resulting in an ion *m/z* 347, which with cleavage of the methylene bridge gives a resonance stable ion *m/z* 181 and is of high intensity (Figure 4). From the structure of the same ion under ESI(+)-MS/MS conditions, a neutral water molecule was eliminated, and ion *m/z* 163 was obtained.

From the mass spectral analysis, we found that the ions *m/z* 167, *m/z* 155, and *m/z* 139 are characteristic, which prove that the structure contains α-pyrone, while the ions *m/z* 249, *m/z* 237, *m/z* 193, *m/z* 181, *m/z* 175, and *m/z* 163 evidence for a specifically substituted phloroglucinol fragment. The same fragmentation pathways undergo the derivatives of arzanol, methylarzanol, and heliarzanol. Fragmentation of methylarzanol by pathway 2 produced a fragment ion *m/z* 169, demonstrating that the structure of the α-pyrone fragment contained one methyl group (Figures S3 and S4). Under ESI(+) MS/MS conditions, the arzanol derivatives (*m/z* 431) also gave the same characteristic fragment ions for the phloroglucinol fragment (Figures S5 and S6). In heliarzanol, the phloroglucinol fragment is more substituted, and the ions obtained in paths 1 and 2 are *m/z* 293, *m/z* 281, *m/z* 207, *m/z* 195, and *m/z* 177. The ions *m/z* 167, *m/z* 155, and *m/z* 139 confirmed that its structure contained an α-pyrone (Figures S7 and S8). Pyrones helipiron and italipyrone contain the same α-pyrone fragment in their structure. The helipyrone structure contains two α-pyrone fragments linked by a methylene bridge, while the italipyrone structure contains an additional tremetone core, also linked by a methylene bridge. Only the ions *m/z* 167, *m/z* 155, and *m/z* 139 were generated in the fragmentation of helipyrone (Figures S9 and S10). Fragmentation of italipyrone generated ions that were characteristic of the tremetone fragment (*m/z* 235, *m/z* 247) (Figures S11 and S12). In the hexane fraction, we identified a total of 10 components that are representatives of phloroglucinols. The analysis data show that the main components are the arzanol group (Table A1).

MS/MS data revealed that CHCl_3_ extracted tremetones and flavonoid aglycones. From the group of aglycones, we identified nine flavonoids, including methoxyluteolin, quercetin-3-methyl ether, luteolin, naringenin, herbacetin-3-methyl ether, kaempferol-3-methyl ether, pinocembrin, galangin, and galangin-3-methyl ether. From the group of tremetones, we identified bitalin A, gnaphaliol, 3-acetoxy-10-hydroxytremeton, 13-(2-methylpropanoyloxy)toxol, and 3-hydroxy-10-propionyloxytremeton (Figure 3B, Table A1). Tremetones were the major components in the chloroform fraction as demonstrated by HPLC-PDA and UHPLC-MS/MS. The UV spectrum shows that the same compounds absorb at λ_max_ 280 nm.

**Scheme 2:**
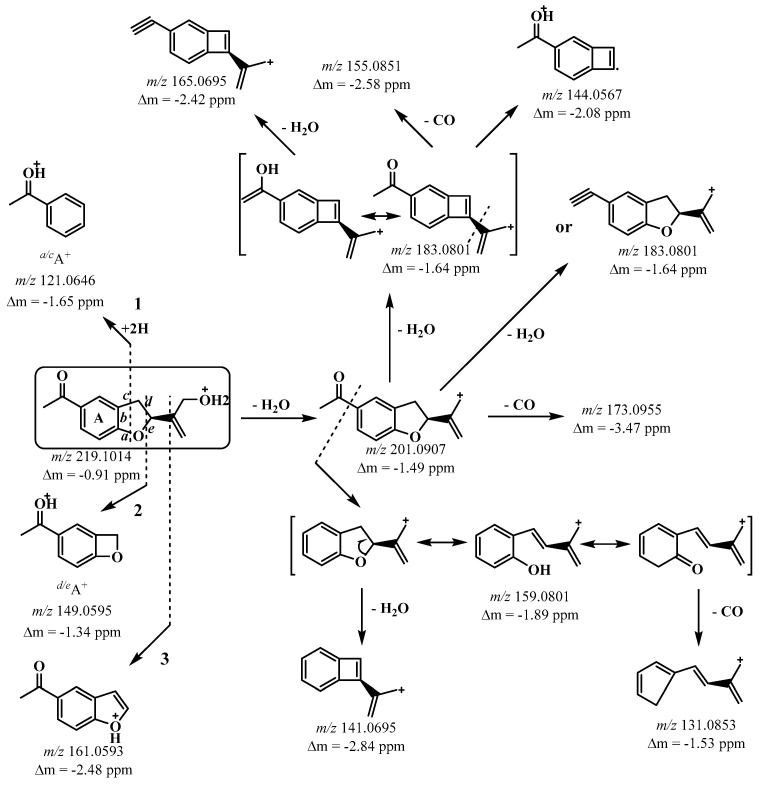
Proposed fragmentation of protonated bitalin A [M+H]^+^.

**Figure 5:**
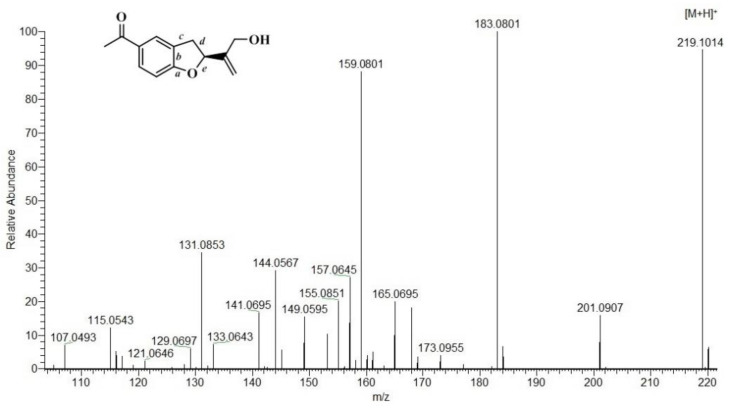
Mass spectrum of bitalin A obtained by positive ion ESI-MS/MS.

The structure of the tremetones contains the same structural fragment, a benzo[*b*]furan ring. A significant difference is in the various substituents associated with tremetone. Several pathways of fragmentation of the tremetone structure originate from here. The main fragmentation pathways are associated with retrocyclization of the *a/c* bonds (pathway 1), *d/e* (pathway 2), as well as cleavage of the C2–C8 bond (pathway 3) (Scheme 2). With increasing C3 substituents, no retrocyclization of the furan ring occurs. Retrocyclization of bitalin A yields two fragments. Path 1 with cleavage of the *a/c* bond (Path 1) yields a fragment ion *^a/c^*A^+^ with *m/z* 121, which corresponds to the structure of acetophenone (Scheme 2). The same ion is of low intensity (Figure 5). A subsequent retrocyclization was performed via pathway 2 with cleavage of the *d/e* bonds, yielding ion *m/z* 149 (*^d/e^*A^+^) (Scheme 2, Figure 5). Path 3 related to cleavage of the C2–C8 bond and leads to the formation of ion *m/z* 161. In addition to the mentioned pathways of fragmentation of bitalin A, rearrangements occur with the release of neutral molecules CO and H_2_O. The bitalin A molecular ion systematically loses three molecules of water, yielding the ions *m/z* 201 [M+H-H_2_O]^+^, *m/z* 183 [M+H-2H_2_O]^+^, and *m/z* 165[M+H-3H_2_O]^+^ (Scheme 2, Figure 5). Ion *m/z* 183 is of high intensity, which is due to the fact that isomeric and resonance stable fragments are obtained (Figure 5). The same ion undergoes parallel loss of CO and a propene fragment to yield the *m/z* 155 and *m/z* 144 ions (Scheme 2, Figure 5). Ion *m/z* 201 loses 42 (C_2_H_2_O) Da and gives rise to resonance stable ion *m/z* 159 [M+H-H_2_O-C_2_H_2_O], which undergoes rearrangement with release of H_2_O and CO. The fragment ions corresponding to [M+H-2H_2_O-C_2_H_2_O] (*m/z* 141) and [M+H-H_2_O-C_2_H_2_O-CO] (*m/z* 131) were obtained.

The retrocyclization of gnaphaliol proceeds by cleavage of the *d/e* bonds, yielding ions *m/z* 149 and *m/z* 163, corresponding to the fragments [*^d/e^*A^+^-OH] and [*^d/e^*A^+^], respectively (Figures S13 and S14). These fragments provide information that the gnaphaliol structure contains an OH group at the C3 position. The gnaphaliol (*m/z* 235) also undergoes rearrangement with release of neutral CO and H_2_O molecules. The molecular ion of gnaphaliol successively loses a molecule of H_2_O and ions *m/z* 217 [M+H-H_2_O]^+^ and *m/z* 199 [M+H-2H_2_O]^+^ are obtained. Ion *m/z* 217 successively undergoes loss of CO and H_2_O and gives rise to fragment ions *m/z* 189 [M+H-H_2_O-CO]^+^ and *m/z* 171 [M+H-2H_2_O-CO]^+^. In addition, the same ion undergoes an additional loss of 42 Da to give an *m/z* 175 [M+H-H_2_O-C_2_H_2_O]^+^ ion. This ion also undergoes rearrangement with release of CO and H_2_O. The fragments *m/z* 157 [M+H-2H_2_O-C_2_H_2_O]^+^ and *m/z* 147 [M+H-H_2_O-C_2_H_2_O-CO]^+^ were obtained. The *m/z* 129 fragment was obtained after loss of H_2_O from the *m/z* 147 ion (Figures S13 and S14).

3-Acetoxy-10-hydroxytremeton, 13-(2-methylpropanoyloxy)toxol, and 3-hydroxy-10-propionyloxytremeton are tremetone derivatives. Gnaphaliol derivatives also undergo rearrangements with release of CO and H_2_O. Cleavage of the C2–C8 bond produced the ions *m/z* 219, *m/z* 127, and *m/z* 113, respectively (Figures S15–S20). Fragmentation of the gnaphaliol derivatives yielded the characteristic ions *m/z* 235, *m/z* 217, *m/z* 189, *m/z* 179, *m/z* 151, and *m/z* 123 (Figures S17–S20).

**Figure 6:**
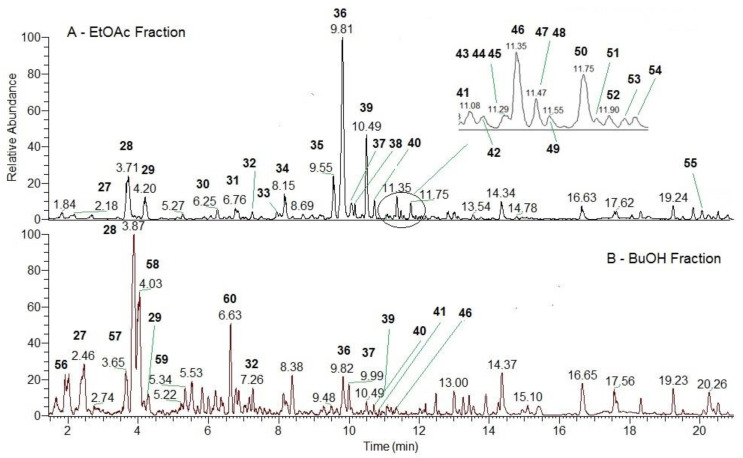
Total ion current (TIC) of phenolic compounds from *H. italicum* in EtOAc fraction (**A**); total ion current (TIC) of phenolic compounds from *H. italicum* in BuOH fraction (**B**). Phenolic acid and flavonoid-glycoside were identified in the EtOAc fraction (**A**). Hydroxyptalid*e*glycoside, c*affeic acid*-glycoside, and quercetin diglycoside (peaks—56, 57, 58, 60) were identified in the BuOH fraction (**B**).

UHPLC-MS/S analysis showed that in the ethyl acetate fraction the main components were mono- and di-caffeoylquinic acids (Figure 6A, Table A1).

The results of the analysis coincide with the data obtained from their UV spectra. In the same fraction, we determined the content of glycosides of myricetin, quercetin, isorhamnetin, and kaempferol. The content of quercetin glycosides is the highest, followed by isorhamnetin glycosides. In the ethyl acetate fraction, we identified 29 components (Figure 6A, Table A1), a large number of which were confirmed by previous studies. In addition, we identified components not previously reported in the polyphenolic composition of *H. italicum*. Identification was made by ESI(−) MS/MS.

Peak 42 with t_R_ 11.25 min (Figure 6A, Table A1) was identified as quercetin acetyl-glycoside. The molecular ion *m/z* 505 lost 204 Da (acetyl-glycoside) in the MS/MS experiment, and an ion *m/z* 301 ([M-H-204]^−^) was produced. The resultant ion 301 split into the quercetin ions *m/z* 179 [^1.2^A^−^], *m/z* 151 [^1.2^A^−^–CO], and *m/z* 107 [^1.2^A^−^–CO–CO_2_].

Peak 43 with t_R_ 11.29 min (Figure 6A, Table A1) was identified as isorhamnetin acetyl-glycoside with molecular ion *m/z* 519. It directly lost 204 Da, resulting in ion *m/z* 314. The same ion loses 15 Da, and an *m/z* 299 ion is obtained.

Peak 45 with t_R_ 11.28 min (Figure 6A, Table A1) was identified as coumaroyl-caffeoylquinic acid with molecular ion *m/z* 499. The molecular ion lost 146 Da, which corresponds to coumaric acid. The product ion *m/z* 353 loses 162 Da (caffeic acid) to give an ion *m/z* 191. Ions *m/z* 179, *m/z* 161, and *m/z* 135 are characteristic of caffeic acid, while ions *m/z* 191 and *m/z* 173 are characteristic of quinic acid.

Peak 48 with t_R_ 11.49 min (Figure 6A, Table A1) was identified as isorhamnetin caffeoyl-glycoside with molecular ion *m/z* 639. It lost caffeic acid and generated ion *m/z* 477 ([M-H-caffeoyl]^−^). The ion *m/z* 477 additionally loses 162 Da and the resulting ion is *m/z* 315 ([M-H-caffeoyl-162]^−^). The same ion (*m/z* 315) fragments into the characteristic ions *m/z* 151 [^1.3^A¯] and *m/z* 107 [^1.3^A¯–CO_2_].

Peak 51 with t_R_ 11.77 min (Figure 6A, Table A1) was identified as quercetin caffeoyl-malonyl-glycoside with molecular ion *m/z* 711. Under ESI(−) MS/MS conditions, the same molecular ion lost a neutral CO_2_ molecule, yielding ion *m/z* 667 [M–H–CO_2_]^−^. This indicates that the structure of the compound contains a fragment with a free carboxyl group. The product ion loses 162 Da (hexose or caffeoyl), and ion 505 [M–H–CO_2_–162]^−^ is obtained. The same ion undergoes the loss of a neutral H_2_O molecule to give ion 487 [M–H–CO_2_–162–H_2_O]^−^. From the mass spectral studies, it is understood that the molecular ion structure contains one unit of caffeic acid, malonic acid, and hexose (glucose and galactose). In the MS/MS experiment, the molecular ion lost 410 Da (caffeoyl-malonyl-glycoside). The resulting ion from the MS/MS experiment was quercetin and its specific fragment ions *m/z* 179 [^1.2^A¯], *m/z* 151 [^1.2^A¯–CO], and *m/z* 107 [^1.2^A¯–CO–CO_2_].

Peak 53 with t_R_ 12.05 min (Figure 6A, Table A1) was identified as isorhamnetin coumaroyl-glycoside with molecular ion *m/z* 623. It lost 308 Da, and the resulting ion was *m/z* 315 ([M-H-coumaroyl-162]^−^).

Peak 54 with t_R_ 12.55 min (Figure 6A, Table A1) was identified as a diCQA-acetyl derivative with molecular ion *m/z* 681. It lost 42 Da, and the resulting ion was *m/z* 639 ([M-H-42]^−^). The product ion loses 124 Da, yielding ion 515. The same ion systematically loses 162 Da (caffeoyl), yielding ions *m/z* 353 and *m/z* 191. Additionally, ions *m/z* 179, *m/z* 161, and *m/z* 135 are characteristic of caffeic acid, while ions *m/z* 191 and *m/z* 173 are of quinic acid.

Polar fractions extract phenolic acids flavonoid-glycosides. Ethyl acetate and butanol as polar solvents possess the ability to extract a large number of polyphenolic components. In general, part of the profile of the BuOH fraction matches the profile of the EtOAc fraction. From both profiles, we found that butanol was a better extractant of mono-caffeoylquinic acids, caffeic acid glycosides, and more polar glycosides (Figure 6B, Table A1).

By UHPLC-MS/S analysis of the fractions, we identified a total of 60 compounds. For the first time, eight new compounds are identified as quercetin 3-*O*-glucuronide, quercetin acetyl-glycoside, isorhamnetin acetyl-glycoside, isorhamnetin caffeoyl-glycoside, quercetin caffeoyl-malonyl-glycoside, isorhamnetin coumaroyl-glycoside, coumaroyl-caffeoylquinic acid, and diCQA-acetyl-derivative, which have not been reported in the polyphenolic composition of *H. italicum* ever before.

### 2.5. Hydrogen Peroxide Scavenging Activity (HPSA)

Reactive oxygen species (ROS) is a term used directly for oxygen-containing free radicals or molecular species capable of generating free radicals. Most of the reactive oxygen species, including superoxide anion (O_2_^.−^), hydrogen peroxide (H_2_O_2_), and hydroxyl radical (^•^OH), are produced as byproducts of aerobic metabolism and increased during infections, inflammatory processes, stress conditions, radiation, and others [44]. ROS damage vital molecules of important biological importance such as phospholipids, proteins, and DNA. This chemical damage is commonly referred to as oxidative stress and is believed to be responsible for numerous health disorders such as cancer, cardiovascular disease, atherosclerosis, and Alzheimer’s disease. [45]. As a result, the sulfur-containing compounds glutathione, cysteine, and ergothioneine play a vital role as endogenous antioxidants in the human body [46]. Therefore, it is necessary to take exogenous antioxidants, which accelerate their inhibition. This, in turn, reduces the aging process and prevents the balance of the internal antioxidant system from being disturbed.

Among ROS, H_2_O_2_ is an important molecule because although it is not toxic by itself, it can be converted into other even more toxic radicals such as ^•^OH by the Fenton reaction or hypochlorous acid by the enzyme myeloperoxidase (MPO, EC 1.11.2.2) [47]. Aside from ROS creation during aerobic cellular metabolism, the inflammatory process also contributes to and accelerates ROS synthesis.

**Figure 7:**
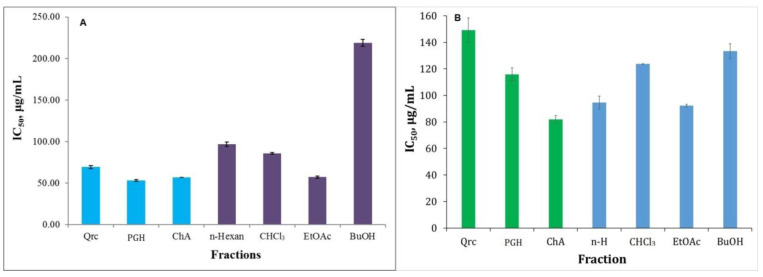
HPSA (**A**) and HRSA (**B**) of the fractionated polyphenolic complex with different polarity solvents obtained from *H. italicum*. Quercetin (Qrc), phloroglucinol (PGH), and chlorogenic acid (ChA) were used as standards. The antioxidant activity results are presented as IC_50_ (µg/mL).

For this reason, inhibition of H_2_O_2_ is very important to prevent the generation of ^•^OH. Therefore, in the present work, research was focused on the harmful effects of hydrogen peroxide. To evaluate the antioxidant activity, we used standards that were selected according to the polyphenolic composition reported in the literature. We compared the results obtained for the antioxidant activity of the individual fractions with the standards quercetin, phloroglucinol, and chlorogenic acid. The HPSA values of the obtained fractions ranged from 57.12 µg/mL to 218.86 µg/mL (Table 2, Figure 7).

The ethyl acetate fraction (57.12 µg/mL) showed the highest antioxidant activity compared to quercetin (69.25 µg/mL) and the other fractions (Table 2, Figure 7A). This is because ethyl acetate as a polar solvent has the ability to isolate polar polyphenols such as CQA and flavonoid glycosides. The results obtained for high HPSA correlated with the high content of CQA (45.82 mg/g Extr) and flavonoids (2.24 mg/g Extr) (Table 1). Apparently, the presence of caffeic acid residue in the CQA structure exhibits a high affinity for H_2_O_2_ deactivation. Despite the nonpolar nature of the hexane and chloroform fractions, they exhibited higher HPSA than the butanol fraction. The analysis of the study proves that phloroglucinols are characterized by high activity. This fact is verified by the structure of the PGH standard, which has the ability to inhibit the harmful action of H_2_O_2_. The HPSA of the fractions decreased in the following order: EtOAc > CHCl_3_ > n-Hexane > BuOH.

### 2.6. Hydroxyl Radical Scavenging Activity (HRSA)

Hydroxyl radicals (^•^OH) also play an important role in the physiological and pathological processes of organisms. Protein carbonylation is associated with oxidation of protein side chains by introducing ketone and aldehyde groups into the protein. Protein carbonylation is of interest because of its association with various diseases [48].

Although H_2_O_2_ and ^•^OH radicals result from oxidative stress, ^•^OH radicals are more reactive. Their inhibition apparently occurs by a mechanism different from that of H_2_O_2_. High HRSA depends on the structure of the compounds. Therefore, the standards are structurally different. In the obtained fractions, the polyphenol composition has a structure close or identical to the standards. In this way, we obtain useful information about which group of compounds have an affinity for ^•^OH radicals.

The results of the analysis show a correlational relationship between structure and activity. Chlorogenic acid as a standard has a high affinity to scavenge ^•^OH radicals, followed by phloroglucinol (Figure 7B). The IC_50_ values of the different fractions ranged from 92.23 µg/mL to 133.31 µg/mL (Table 2). The HRSA data confirmed that the ethyl acetate and hexane fractions exhibited the highest activity (Figure 7B). This proves that chlorogenic compounds and phloroglucinols have a high affinity for scavenging ^•^OH radicals.

### 2.7. Metal-Chelating Activity

**Figure 8:**
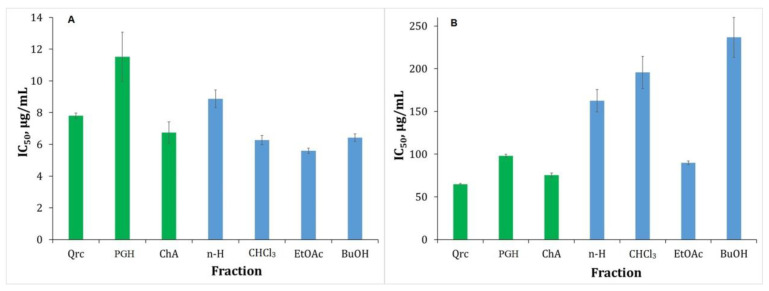
MChA (**A**) and NOSA (**B**) of the fractionated polyphenolic complex with different polarity solvents obtained from *H. italicum*. Quercetin (Qrc), phloroglucinol (PGH), and chlorogenic acid (ChA) were used as standards. The antioxidant activity results are presented as IC_50_ (µg/mL).

Iron ions (Fe^2+^) are well known for their increased propensity for the Fenton reaction, but this also makes them one of the most important pro-oxidants involved in lipid peroxidation, i.e., oxidation of lipids leading to cell membrane damage. This, in turn, accelerates the aging process. Inhibiting the catalytic action of Fe^2+^ ions occurs through their chelation and is one of the existing antioxidant methods. Chelating activity on ferrous iron is an important step in preventing lipid peroxidation [49]. Therefore, we investigated the ability of the fractions to form chelate complexes with Fe^2+^. The concentration gradient of the fractions affects the chelating activity. As the concentration of the fractions increases, their ability to chelate Fe^2+^ increases. The metal-chelating activity of the investigated fractions varies in the range 5.60–8.87 µg/mL (Table 2). In general, all fractions exhibit high chelating activity. In this context, we compare which components show greater activity. From the analysis, we found that the ethyl acetate fraction showed significant metal-chelating activity due to the presence of CQA and the flavonoid-glycosides in it (Figure 8A). This is because the structure of mono- and di-CQAs and flavonoid-glycosides contain free non-sterically hindered OH groups. The hexane fraction is characterized by the lowest activity. This is also explained by the PGH standard. Moreover, in the structure of arzanols, OH groups are sterically hindered by nonpolar substituents. On the other hand, it can be explained by the fact that phloroglucinols exhibit low chelating activity.

### 2.8. Nitric Oxide Scavenging Activity (NOSA)

NO is an essential bioregulatory molecule required for several physiological processes such as neural signal transmission, immune response, vasodilation, and blood pressure control. However, an increase in the level of NO can lead to several pathological conditions, including cancer. NO is generated from the terminal guanido nitrogen atom of L-arginine by various NADPH-dependent enzymes called NO synthases (NOS).

NO does not interact directly with bioorganic macromolecules such as DNA or proteins. However, under aerobic conditions, the NO molecule is very unstable and reacts with oxygen to produce intermediates such as NO_2_, N_2_O_4_, and N_3_O_4_, and when it reacts with superoxide, stable products such as nitrate, nitrite, and peroxynitrite are generated. The products obtained as a result of nitrosative stress are highly genotoxic [50,51].

In the present study, the same trend as in HRSA was observed. The ethyl acetate and hexane fractions were characterized by high NOSA (Figure 8B). Quercetin and chlorogenic acid standards are also characterized by high activity. The high activity of the ethyl acetate fraction is due to the high content of chlorogenic acids and quercetin flavonoids (Table 1). Phloroglucinol is also characterized by high activity. The high activity of the hexane fraction is due to the content of phloroglucinols. In addition, a good correlation relationship was observed between NOSA and the content of phenolics, tannins, flavonoids, and CQAs, with the correlation coefficient ranging (r) from 0.7176 to 0.9684.

In the present work, we investigated the antioxidant activity assessed as HPSA, HRSA, and NOSA. The first two methods are responsible for oxidative stress and the third for nitrosative stress. In general, the investigated fractions showed very good antioxidant activity. It is due to the components contained in them.

### 2.9. Inhibition of Albumin Denaturation (IAD)

**Figure 9:**
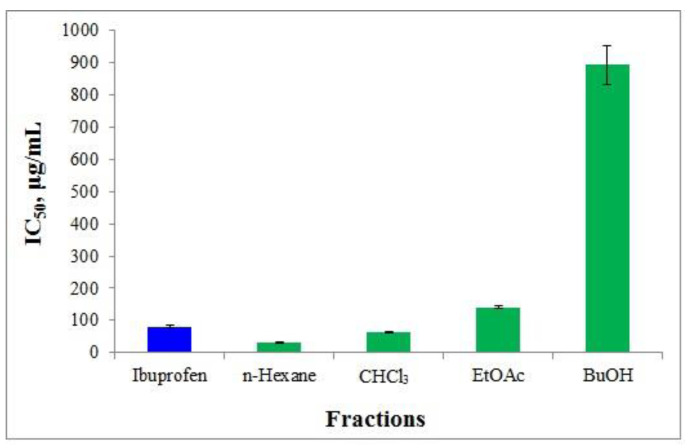
IAD by *H. italicum* fractionated polyphenolic complex solvents with different polarities. As a control, ibuprofen (Ibu) was used. The results are presented as IC_50_ (µg/mL).

Inflammation is the reaction of living tissues to harm. It consists of a complicated combination of enzyme-activated events such as mediator release, fluid extravasation, cell movement, tissue destruction, and repair [52]. Protein denaturation has also been linked to rheumatoid arthritis inflammation. Several anti-inflammatory medications have been found to have a dose-dependent ability to prevent protein denaturation [53]. The obtained H. italicum fractions were evaluated for albumin denaturation inhibition. This approach determines the extent to which albumin can be protected against denaturation by heating. Human albumin was employed for this purpose. Figure 9 depicts the inhibition percentages of the various H. italicum fractions.

The results of the study are presented as IC_50_. Since ibuprofen has proven anti-inflammatory properties, we decided to use it as a reference to compare the activity of different fractions of *H. italicum*. The IC_50_ of ibuprofen estimated as IAD was 81.50 μg/mL (Table 2, Figure 9). The obtained results showed that the IC_50_ of the different fractions ranged from 30.48 μg/mL to 892.21 μg/mL (Table 2, Figure 9).

Comparing with the standard and evaluating the degree of albumin protection by polarity of the fractions, the hexane and chloroform fractions show high activity. The high activity of the hexane fraction is most likely due to the presence of the phloroglucinols arzanol, methylarzanol, heliarzanol, and arzanol derivatives.

For the stability of albumin, the hydrophobic centers of subdomains IIA and IIIA play an important key role, known as Sudlow I and Sudlow II allosteric centers. Due to the hydrophobic nature of the allosteric center of Sudlow I, the drug mainly forms hydrophobic interactions with the amino acid residues involved in the structure of this center Phe211, Trp214, Leu219, Phe223, Leu234, Leu238, Leu260, Ile264, and Ile290 [54]. This is precisely the reason why the arzanol group exhibits high activity. The tremetones contained in the CHCl_3_ fraction also showed high activity. This fact can be explained by the structure, or regardless of the low content of aglycones, they can also show a synergistic effect.

### 2.10. Docking of the Three Ligands (Arzanol and R/S-Bitalin A) Using AutoDock Vina- Molecular Dynamics Study for Validation of the Binding of Compounds to Albumin

The affinity of arzanol and bitalin A to the human serum albumin was studied in a molecular docking study, targeting the most important four sites of albumin: Sudlow 1, Sudlow 2, Site 3, and Cleft. The results of the molecular docking study to the four sites of albumin are presented in Table 3.

The analysis of the affinity values of the studied compounds for albumin sites indicates that arzanol has better affinity for three of the albumin sites, when compared to both enantiomers of bitalin A for the Sudlow site 1 (−7.9 kcal/mol), for the Sudlow site 2 (−9.6 kcal/mol), and for the Site 3 (−9.0 kcal/mol). 

The highest difference between affinities of the studied compounds is identified for the Sudlow 2 site, while for the site 3 the affinity or arzanol (−9.0 kcal/mol) is close to the (*S*)-bitalin A (−8.8 kcal/mol). Overall, for the all sites, (*S*)-bitalin A exhibits only slightly higher affinity than (*R*)-bitalin A, being almost identical, except for site 3 where a bigger difference was identified, as presented before.

On the other hand, both bitalin A enantiomers exhibit a higher affinity for the cleft site (−7.4 kcal/mol and −7.5 kcal/mol) when compared to arzanol (−7.0 kcal/mol).

The top binding conformation of each compound in any of the studied sites was subjected to a molecular dynamic study, after generating the respective complexes with albumin, as follows: arzanol in the site Sudlow 2 and both enantiomers of bitalin A in the site 3. 

The poses of the highlighted affinities to the respective sites were used in the molecular dynamic study.

**Figure 10:**
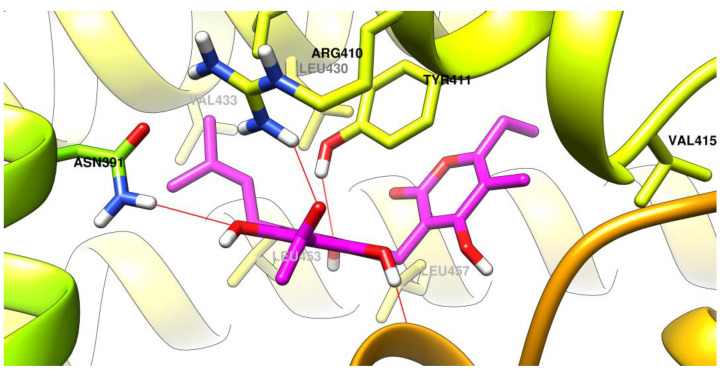
The best binding conformation of arzanol (carbon atoms in magenta) in the site Sudlow 2 of albumin.

**Figure 11:**
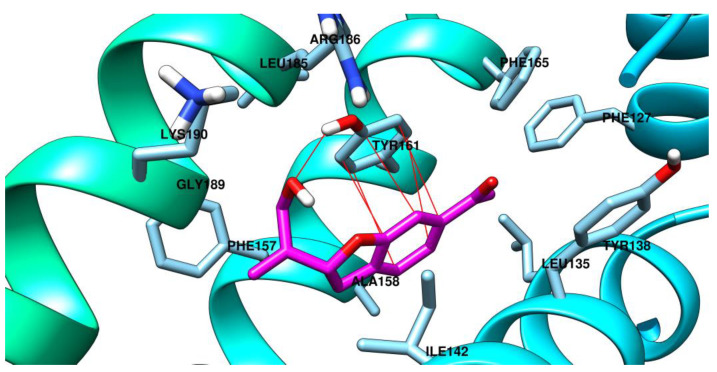
The best binding conformation of (*R*)-bitalin A (carbon atoms in magenta) in the site 3 of albumin.

**Figure 12:**
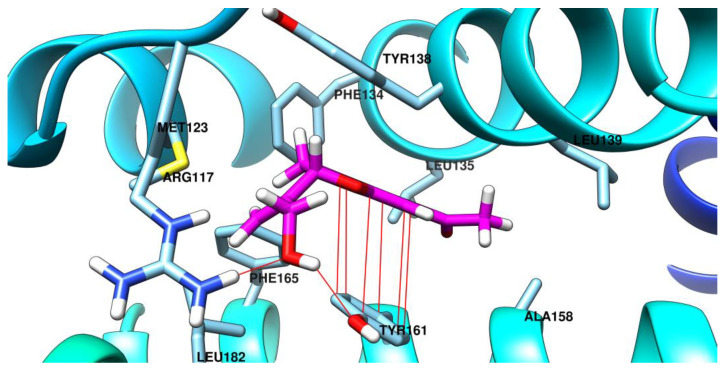
The best binding conformation of (*S*)-bitalin A (carbon atoms in magenta) in the site 3 of albumin.

The interactions between the studied ligands and sites of albumin are graphically depicted as follows: arzanol in the site Sudlow 2 (Figure 10), (*R*)-bitalin A in the site 3 (Figure 11), and (*S*)-bitalin A in the site 3 (Figure 12).

Two phenol oxygen atoms from the phenol groups of arzanol phloroglucinol moiety are involved in hydrogen bonds as acceptors with Asn391 and Tyr411. The third phenol group is predicted to be involved in a hydrogen bond as donor with the peptide bond Phe488-Ser489. The oxygen atom from the exocyclic ketone is predicted to have an ion-dipole interaction with the positively charged Arg410. The lipophilic 3-methylbut-2-ene bound to the phloroglucinol accommodates in a lipophilic region of the binding site comprised of sidechains of Val433, Leu453, Leu407, Leu430, and Leu457. The 2-pyranone substituted with lipophilic ethyl and methyl groups fits in another lipophilic region of the binding site comprised of sidechains of Lys414, Val415, and Val418.

(R)-Bitalin A is predicted to have the most important interaction with Tyr161 sidechain in two ways: one by π–π interaction with the benzene ring from the 2,3-dihydrobenzofurane system and another as acceptor of a hydrogen bond at the oxygen atom from the prop-2-en-1-ol fragment. Other interactions predicted for (R)-bitalin A in the site 3 of albumin are hydrophobic ones, with Phe157, Ala158, Ile142, Leu185, Phe165, and Phe127.

The molecule is mainly involved in hydrophobic interactions with the amino acid sidechains from the site 3 of albumin. The methyl from the acetyl fragment is found near the hydrophobic sidechain of Ala158 and Leu139, while the prop-2-en-1-ol fragment is accommodated near other hydrophobic residues, such as Leu182, Phe165, Met128, and Phe134. The alcoholic OH of (*S*)-bitalin A from the prop-2-en-1-ol fragment is involved in an ion-dipole interaction with the positively charged sidechain of Arg117 and in a hydrogen bond as donor with phenol OH from Tyr161. The benzene ring from the 2,3-dihydrobenzofurane system is involved in π–π interaction with sidechain of Tyr161.

The stability of the albumin complexes with the best binding conformation of each ligand resulted from the molecular docking study was evaluated using a 100 ns of molecular dynamics simulation. As presented in the results of the molecular docking, the best binding conformations of each compound to be subjected to the molecular dynamics study were chosen as follows: arzanol in the site Sudlow 2, (*R*)-bitalin A in the site 3, and (*S*)-bitalin A in the site 3.

The stability of the protein–ligand simulated systems in the molecular dynamics study was expressed by calculating the average root-mean-square deviation (RMSD) of the atoms from the backbone of the protein, the average root-mean-square deviation (RMSD) of the heavy atoms of ligands, the radius of gyration (RG) of the protein, and the number hydrogen bonds between the ligand and the protein. Analysis of evolution of the simulated complexes of albumin with arzanol, (*R*)-bitalin A, and (*S*)-bitalin A indicates that all three complexes are stable during the 100 ns simulation time. An overview of the results of the molecular dynamics study is presented in Table 4, presenting the RMSD of the backbone of the protein, the RMSD of the heavy atoms of ligands, the RG of the protein, and the number of hydrogen bonds between the protein and ligands in time. A detailed evolution of the simulated systems regarding the respective parameters depicted as charts is found in Figures S21–S24.

In terms of the fluctuations of the backbone of the protein, all three compounds exhibit a stabilization of the albumin compared to the apo albumin, the RMSD of the backbone of the protein being smaller when complexed with all three compounds. When analyzing the behavior of ligands in the sites where they were bound, a very strong inverse correlation between the RMSD of their coordinates and the number of hydrogen bonds between the ligand and the protein can be seen. (*S*)-bitalin A has the highest average hydrogen bonds with the protein from the current series of compounds (0.33/ns) and the lowest RMSD (0.24 nm). Arzanol, which has an intermediate number of hydrogen bonds (0.11/ns), has the intermediate fluctuation in the site expressed as RMSD (0.36 nm). (*R*)-bitalin A, which has the lowest hydrogen bonding from the present series (0.01/ns), has the highest fluctuation in the site expressed as RMSD (0.48 nm).

Arzanol exhibited the strongest binding to the albumin compared to the two enantiomers of bitalin A. All three resulting complexes are stable in time. After complexation with the three compounds, the albumin backbone fluctuates lower than the apo form, with similar values for the three complexes with each compound.

The influence of stereochemistry in the binding of compounds to albumin was strongly identified in the case of bitalin A enantiomers. In all four tested sites, (*S*)-bitalin A exhibited a stronger binding than (*R*)-bitalin A, expressed as variation of Gibbs free energy. Strictly about their binding in the site 3 of albumin, the difference in the spatial orientation of the prop-2-en-1-ol fragment influences the affinity to the protein and the stability of the resulted complexes in time. (*S*)-Bitalin A being stronger bound through more hydrogen bonds and strong polar interactions to the protein compared to (*R*)-bitalin A, its position is more stable inside the pocket.

### 2.11. Anti-Tryptic Activity (ATA)

**Figure 13:**
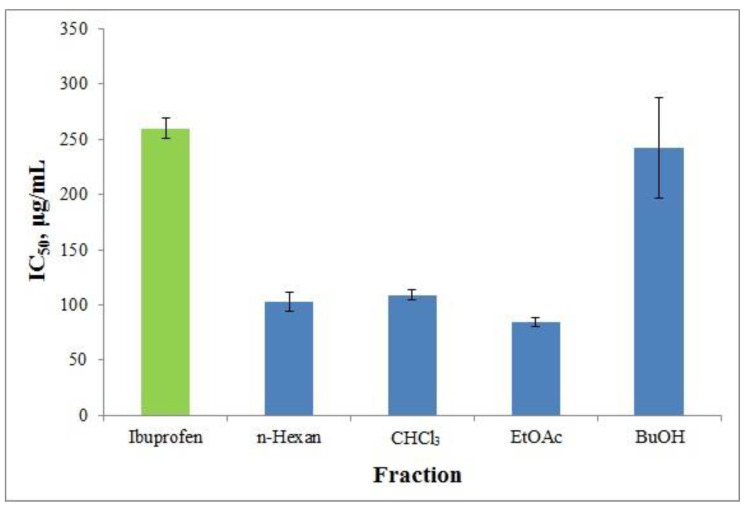
ATA of the fractionated polyphenolic complex with different polarity solvents gained from *H. italicum*. Ibuprofen (Ibu) was used as a control. The results are given as IC_50_ (µg/mL).

Proteinases have been identified as the root cause of arthritic disorders. Neutrophils are known to be a major source of proteinases, with several serine proteinases found in their lysosomal granules. Proteinase, which is found in leukocytes, is known to play a major part in tissue damage during inflammatory reactions, and proteinase inhibitors give a high level of protection [53,55]. *In vitro* anti-arthritic activity was evaluated as anti-tryptic activity [55]. This study aims to investigate the suppression of the serine proteinase trypsin, another enzyme that serves a similar function. Anti-tryptic activity was tested in the resultant fractions from *H. italicum* (Table 2, Figure 13). ATA IC_50_ values ranged from 84.19 μg/mL to 242.23 μg/mL.

The results of the study revealed that, with the exception of the butanol fraction, the polar and nonpolar fractions had higher inhibitory efficacy than ibuprofen. The maximum ATA (84.19 g/mL) was found in the ethyl acetate fraction. High activity is a characteristic of the chlorogenic acids that were extracted from ethyl acetate. Additionally, phloroglucinols exhibit greater activity than profens. The structure of chlorogenic chains and phloroglucinols is consistent with the active core of the enzyme, which accounts for the compounds’ action.

### 2.12. UV-B Protection

**Figure 14:**
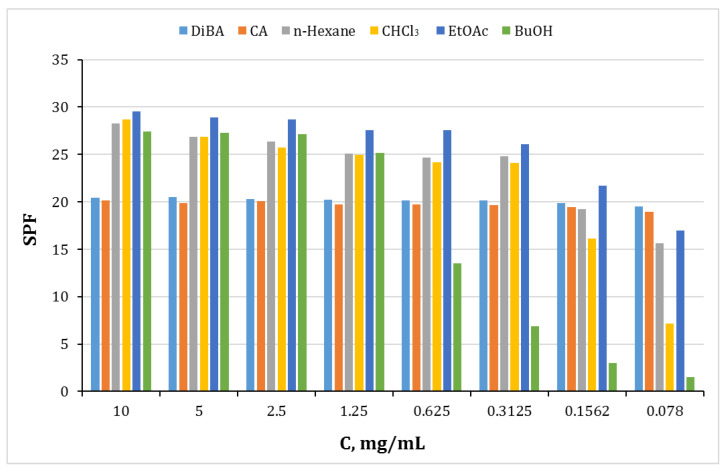
Sun protection factor (SPF) study data of the fractionated polyphenolic complex with different polar solvents obtained from *H. italicum*. Dibenzylidene acetone (DiBA) and cinnamic acid (CA) were used as standards.

The method described by Mansoor in the literature is widely used for in vitro evaluation of sun protection factor (SPF), which proves a good correlation as a substitute for in vivo tests, as it relates to the absorption of the substance with the erythematous effect of radiation and the light intensity at wavelengths from 290 to 320 nm (UV-B region of the spectrum). Figure 14 shows the sun protection factor of the different fractions. Absorption of UV-B rays is a function of SPF. As the SPF values increase, the percentage of absorbed UV-B radiation increases (Figure 14), respectively; this leads to a decrease in the erythematous effect.

In this study, we used cinnamic acid and dibenzylidene acetone as reference standards for SPF evaluation. The SPF values in the investigated concentration range do not statistically change. This means that at low concentrations, a large effect is achieved, i.e., at 78 µg, SPF 20 is achieved, which means 95% UV-B protection [56].

## 3. Materials and Methods

### 3.1. Chemicals and Reagents

Chromatographic-grade methanol (VWR, Vienna, Austria) was used for HPLC analysis. Water for HPLC was obtained using a Millipore purifier (Millipore, Burlington, MA, USA). Hexane, chloroform, butanol, ethyl acetate, ethanol, acetonitrile, ibuprofen, potassium dihydrogen phosphate, dipotassium hydrogen phosphate, sodium chloride, potassium chloride, sodium carbonate, dibenzylidene acetone, cinnamic acid, sodium salicylate, ferrous sulfate, ferrous dichloride, sodium nitrite, sulfanilamide, phosphoric acid, naphthylethylenediamine dihydrochloride, ferrozine, sodium nitroprusside, chlorogenic acid, phloroglucinol, hydrogen peroxide, ascorbic acid, quercetin, trypsin, Tris-HCl buffer, Folin–Ciocalteu’s reagent, aluminum trichloride, EDTA, sodium hydroxide, formic acid (FA), and perchloric acid were supplied by Sigma-Aldrich Chemie GmbH, Buchs, SG, Switzerland. Human albumin 20%—BB, 200 g/L was purchased from BB-NCIPD Ltd., Sofia, Bulgaria.

### 3.2. Plant Material

We used plant material from *H. italicum* cultivated in the area of the city of Kazanlak (Bulgaria). The plant was harvested in full bloom in 2018. The plant material was provided by Chief Assistant Professor Silvia Mollova, Ph.D. (Institute of Rose and Aromatic Plants—Kazanlak, Bulgaria). It was air-dried and dried in the shade at room temperature. The dry plant material was ground into a powder and stored in a paper bag at room temperature.

### 3.3. Fractionation of Polyphenols with Solvents of Different Polarity

The fractionation of polyphenols with different polarity solvents was described by Bojilov and co-workers [57]. Finely ground pith plant material (10 g) was weighed, transferred to a 500 mL Erlenmeyer flask with a stopper, and extracted with 500 mL methanol for 40 min in an ultrasonic bath. The extract was filtered through a sintered glass filter under vacuum, and then the filtrate was evaporated to dryness. The dry residue was weighed and then suspended in 100 mL of distilled water. The aqueous layer was fractionated by liquid–liquid extraction—sequentially with hexane (10 × 30 mL), chloroform (10 × 30 mL), ethyl acetate (10 × 30 mL), and butanol (10 × 30 mL). The resulting fractions were evaporated to dryness using a rotary vacuum evaporator and weighed. They were then dissolved in methanol, the concentration of each fraction being 10 mg/mL. The obtained fractions were tested *in vitro* for their biological activity and analyzed by HPLC-PDA and UHPLC-MS.

### 3.4. Determination of Total Phenolic Content (TPC)

The total phenolic content in crude extracts was determined with a colorimetric method using Folin–Ciocalteau’s reagent [58] with slight modifications [59]. Calibration curve was achieved using a standard ethanolic solution of gallic acid at concentrations between 50 and 1000 µg/mL. Briefly, 100 µL of extract or gallic acid standard was mixed with 2.4 mL distilled water, 500 µL of 0.2 M Folin–Ciocalteu’s reagent, and 2 mL of 7.5% sodium carbonate (Na_2_CO_3_) solution. The tested samples were incubated for 2 h in the dark at room temperature. The absorbance of the samples was measured at 765 nm with a spectrophotometer (Camspec M508, Leeds, UK) using a blank sample. The total phenolic content was expressed as mg gallic acid equivalent per gram extract (mg GAE/g Extr) based on the calibration curve.

### 3.5. Determination of Total Tannin Content (TTC)

The total tannin content (TTC) in the different fractions was determined spectrophotometrically using the Folin–Ciocalteu method [60]. To determine the total tannin content, we used standard solutions of tannic acid with a concentration of 10 μg/mL to 1000 μg/mL. The analysis was carried out as follows: in a 10 mL volumetric flask, 100 μL of tannic acid, fraction or standard, 7.5 mL of distilled water, 500 μL of 2M Folin–Ciocalteu’s reagent, and 1 mL of 35% sodium carbonate solution (Na_2_CO_3_) were added, and the flask was topped up with distilled water up to the mark. Test samples were mixed and incubated for 30 min in the dark at room temperature. The absorbance of the samples was measured at 700 nm with a spectrophotometer (Camspec M508, Leeds, UK) against a blank. Total tannin content is expressed as mg tannic acid equivalent per gram of extract (mg TAE/g Extr).

### 3.6. Determination of Total Flavonoid Content (TFC)

The total flavonoid (TFC) content of the various fractions was measured using a spectrophotometric approach that was slightly modified from Zhishen’s method. We used standard solutions of quercetin ranging in concentration from 10 µg/mL to 1000 µg/mL to estimate the total flavonoid content. The analysis was carried out as follows: 0.25 mL of quercetin fraction or standard, 2.5 mL distilled water, and 0.15 mL NaNO_2_ (5%) were added to a test tube. After 5 min of vigorous stirring, 0.5 mL of AlCl_3_ (10%) was added. After 6 min, 1.6 mL of 1M NaOH was added. After vortexing the mixture, the absorbance of the samples was measured at 510 nm with a spectrophotometer (Camspec M508, Leeds, UK). The total flavonoid concentration is given in milligrams of quercetin equivalent per gram of extract (mg QE/g Extr) [61].

### 3.7. Analysis of Polyphenols by HPLC-PDA

We used reversed-phase HPLC to separate the components of the polyphenolic complex. For the purposes of the study, we used a Knauer liquid chromatograph with a Smartline Manager 5000 low-pressure mixer, consisting of a Smartline 1000 quaternary pump and a detector with a photodiode matrix—PDA 2800 (Knauer, Berlin, Germany).

Method conditions were optimized using a Kromasil column (150 × 4.6 mm i.d., 5 µm particle size (Supelco, Bellefonte, PA, USA) and mobile phase A—2% FA/H_2_O: 2% FA/AcN = 95:5; mobile phase B—2% FA/H_2_O: 2% FA/AcN = 10:90. Elution was performed with a multistep gradient: 0–15 min, 100%—90% A (0–10% B); 15–35 min, 70% A (30% B); 35–45 min 55% A (45% B); 45–50 min 0% A (100% B). The mobile phase speed was 1.0 mL/min, and the sample injection volume was 20 µL. Maximum separation of chlorogenic acids is attained when these chromatographic conditions are used.

The polyphenols were monitored at 280 nm, 320 nm, 340 nm, and 352 nm. The identification of the chromatographic peaks was confirmed by comparison of the retention times of the samples with those of the standard compounds. The ultraviolet spectral characteristics of the eluted peaks scanned with a photodiode matrix detector (λ = 200–400 nm) were compared with those of the standard compounds. To identify those peaks for which no reference standards were available, the chromatographic characteristics were compared to literature retention times. Dicaffeoylquinic acids were identified by overlaying the chromatographic profiles of the sample with that of green coffee. The use of green coffee extract is called a “surrogate standard,” a technique pioneered by Clifford [62].

### 3.8. Identification of Flavonoids by Orbitrap UHPLC-MS/MS

Separation was achieved on an UHPLC system Dionex Ultimate 3000RSLC (Q-Exactive, Thermo Fisher Scientific, Waltham, MA, USA) with reversed-phase column Kromasil EternityXT C18 (1.8 μm, 2.1 × 100 mm) maintained at 40 °C. The binary mobile phase consisted of A: 0.1% formic acid in water and B: 0.1% formic acid in acetonitrile. The run time was 29.5 min. The following gradient was used: the mobile phase was held at 5% B for 0.5 min, gradually turned to 10% B over 3.5 min, increased gradually to 15% B over 2 min, held at 15% B over 2 min, increased gradually to 35% B over 4 min, held at 35% B over 3 min, increased gradually to 60% B over 5 min, held at 60% B over 3 min, increased gradually to 95% over 4.5 min, and finally held at 95% B over 2 min. The system was then turned to the initial condition of 5% B and equilibrated over 4 min. The flow rate and the injection volume were set to 300 μL/min and 2 μL, respectively.

Mass analyses were carried out on a Q Exactive Plus mass spectrometer (Thermo Fisher Scientific, Inc.) equipped with a heated electrospray ionization (HESI-II) probe (ThermoScientific). The tune parameters for positive mode were as follows: spray voltage +3.5 kV; sheath gas flow rate 36 arbitrary units (a.u.); auxiliary gas flow rate 11 a.u.; capillary temperature 320 °C; probe heater temperature 320 °C; and S-lens RF level 50. The negative mode tuning settings were as follows: spray voltage −2.5 kV; sheath gas flow rate 38 arbitrary units (a.u.); auxiliary gas flow rate 12 a.u.; capillary temperature 320 °C; probe heater temperature 320 °C; and S-lens RF level 50. In both positive and negative mode, the Full-MS/DDMS2 (Top5) experiment was employed. Full MS scans were collected at *m/z* range 150–1500 at a resolution of 70,000 (at *m/z* 200); automatic gain control (AGC) target was 3 × 10^6^; and maximum inject time (IT) was 100 ms. The following instrument settings were used for DD-MS2 scans: resolution 17,500, AGC target 1 × 10^5^, maximum IT 50 ms, isolation window 2.0 *m/z*, stepped normalized collision energy (NCE) 20, 40, 60 (for negative mode) and 10, 30, 60 (for positive mode). ThermoScientific’s Xcalibur 4.0 software was used for data collecting and processing.

### 3.9. Methods for Investigation of Biological Activity

#### 3.9.1. Hydrogen Peroxide Scavenging Activity (HPSA)

The Manolov et al. approach was used to evaluate a capacity to scavenge hydrogen peroxide [63]. A 43 mM solution of H_2_O_2_ was prepared in potassium phosphate buffer solution (0.2 M, pH 7.4). The analysis of the samples was carried out as follows: in test tubes, 0.6 mL H_2_O_2_ (43 mM), 1 mL sample/standard with different concentrations (20–1000 µg/mL), and 2.4 mL potassium phosphate buffer solution were mixed. The mixture was stirred and incubated in the dark for 10 min at 37 °C. Absorbance was measured at 230 nm with a spectrophotometer (Camspec M508, Leeds, UK) against a blank solution containing phosphate buffer and H_2_O_2_ without the sample. Quercetin, phloroglucinol, and chlorogenic acid were used as standards. The percentage HPSA of the samples was evaluated by comparing with a blank sample and calculated using the following formula:

(1) $$I,\%(HPSA)=\left[\frac{A_{blank}-(A_{TS}-A_{CS})}{A_{blank}}\right]\times 100$$

where *A_blank_* is the absorbance of the blank sample, *A_CS_* is the absorbance of the control sample, and *A_TS_* is the absorbance of the test sample.

#### 3.9.2. Hydroxyl radical scavenging activity (HRSA)

Hydroxyl radical scavenging activities of different fractions of *H. italicum* were determined according to the method described by Guo [64]. The scavenging of hydroxyl radicals was done as follows: 0.3 mL sodium salicylate (20 mM), 1 mL FeSO_4_ (1.5 mM), 1 mL sample/standard with different concentrations (20–1000 µg/mL), and 0.7 mL H_2_O_2_ (6 mM). They were mixed immediately, and then the reaction tubes were put in a 37 °C water bath for 1 h. The absorbance of the mixture was recorded at 510 nm against a blank. We used standards with proven high antioxidant activity such as quercetin, phloroglucinol, and chlorogenic acid. The hydroxyl radical scavenging ability was calculated as follows:

(2) $$I,\%(HRSA)=\left[\frac{A_{blank}-A_{sample}}{A_{blank}}\right]\times 100$$

where *A_blank_* is the absorbance without samples and *A_sample_* the absorbance in the presence of the samples of different fractions from *H. italicum*.

#### 3.9.3. Metal-Chelating Activity on (MChA)

The ability of the different fractions to chelate Fe^2+^ was evaluated according to the method reported by Sirin et al. The reaction mixture contains 1 mL sample/standard (in methanol), 3.7 mL methanol, and 0.1 mL FeCl_2_ (2 mmol/L). The reaction began when 0.2 mL of ferrozine (5 mmol/L) was added. After being vortexed, the reaction mixture was kept at room temperature for 10 min. After reaching equilibrium in the mixture, the absorbance of the sample was measured at 562 nm on a UV-VIS spectrophotometer (Camspec M508, Leeds, UK). The blank was prepared as the test sample, but methanol was added instead of the sample/standard. Results were reported as percent inhibition of ferrozine Fe^2+^ complex formation with the sample. The mean IC_50_ value was estimated based on three replicates. Ethylenediaminetetraacetic acid (EDTA)–sodium (Na) (0.5 mg/mL) served as a positive control [49].

#### 3.9.4. Nitric Oxide Scavenging Activity (NOSA)

NO inhibition was measured according to the method of Marcocci [65]. Sodium nitroprusside (SNP) was used as the source for generating NO. The SNP solution (5 mM) was prepared in phosphate-buffered saline (PBS, 0.2 M, pH 7.4). Briefly, the reaction mixture containing 0.5 mL SNP (5 mM) and 1 mL sample/standard with different concentrations (15–1000 μg/mL) was incubated at 25 °C for 180 min. At the end of the reaction time, an equal amount of Griess reagent (1% sulfanilamide in 2% phosphoric acid and 0.1% naphthylethylenediamine dihydrochloride) was added. The absorbance of the chromophore (purple azo dye) formed during the diazotization of nitrite ions with sulfanilamide and subsequent coupling with naphthylethylenediamine dihydrochloride was measured at 546 nm. Quercetin, phloroglucinol, and chlorogenic acid were used as standards. The NO uptake capacity was calculated as follows:

(3) $$I,\%(NOSA)=\left[\frac{A_{blank}-A_{sample}}{A_{blank}}\right]\times 100$$

where *A_blank_* is the absorbance of a blank, and *A_sample_* is the absorbance of the test sample.

#### 3.9.5. Inhibition of Albumin Denaturation (IAD)

Inhibition of albumin denaturation (IAD) was used to measure anti-inflammatory efficacy *in vitro*. The analysis was carried out using the Manolov approach [66]. The experiment was carried out using human albumin. In distilled water (pH 7.4), an albumin (1%) solution was produced. The tested compounds/standards were first dissolved in PBS, resulting in a stock solution with a final concentration of 1000 μg/mL. Then, in PBS, a series of working solutions with varying concentrations (20–500 μg/mL) were produced. The reaction mixture contained 2 mL of varied concentrations of test sample/standard and 1 mL of albumin (1%). The mixture was incubated at 37 °C for 15 min before being heated in a water bath at 70 °C for 15 min. The turbidity was measured at 660 nm with a spectrophotometer (Camspec M508, Leeds, UK) after cooling. As a control, ibuprofen was employed. The experiment was conducted three times. The inhibition of albumin denaturation (IAD) was calculated as a percentage of the control. The control sample was albumin dissolved in distilled water at the same concentration.

(4) $$\%IAD=\left[\frac{A_{blank}-A_{sample}}{A_{blank}}\right]\times 100$$

#### 3.9.6. In Silico Evaluation of the Affinity of Arzanol and Bitalin A to a Human Serum Albumin

The affinity of arzanol, (*R*)-bitalin A, and (*S*)-bitalin A to the human serum albumin was evaluated according to the previously reported protocol in a molecular docking study [67]. The stability of the predicted complexes between human serum albumin and the three compounds was evaluated further using a molecular dynamics study, according to the previously reported protocol [67,68].

The molecular docking of arzanol, (*R*)-bitalin A, and (*S*)-bitalin A was performed targeting human serum albumin (HSA) macromolecule using AutoDock Vina 1.1.2 running under Microsoft Windows 10 [69,70]. The protein was taken from the Protein Data Bank [71] (entry code 7JWN). The preparation of the ligands and macromolecules as target was performed according to the standard procedure, previously reported by our group [70,72,73,74]. The targeted sites for binding of the three compounds were chosen according to the literature, being the most important sites in binding: Sudlow site I (subdomain IIA), Sudlow II (subdomain IIIA), site III, and cleft. Other sites of albumin have a marginal role of binding exogenous molecules [75,76]. The search space was set as a cube with sides equal to 20 for each site. The cartesian coordinates of the center of the searching cubes were set as follows: for Sudlow 1 x = 30.62, y = 25.50, z = 12.43, for Sudlow 2 x = 5.95, y = 18.22, z = 21.06, for site 3 x = 30.15, y = 26.98, z = 37.99, and for cleft site x = 20.89, y = 21.74, z = 22.43. The visualization of the results of the molecular docking study was performed using Chimera 1.10.2 [77].

To evaluate the stability of the complexes of the three compounds with albumin predicted in the molecular docking study, using the best binding conformation of each compound in any of the albumin sites, three complexes of albumin were constructed and were subjected to a molecular dynamics study for 100 ns using GROMACS 2023 [78] working under Debian 11 with CUDA 12 and CHARMM36 force field [79]. Ligands parametrization was made using the CgenFF server [79]. The systems were placed in an orthorhombic box with 1 nm gap at the sides and was filled using the TIP3P solvation water [80]. The simulated systems were constructed, neutralized using sodium ions and relaxed by energy minimization (5000 steps using the steepest descent method and the convergence was reached until the maximum force < 1000 KJ mol^−1^ nm^−1^), equilibrated at NVT and NPT ensembles at 300 K for 100 ps, according to the previous works reported, and the simulations were run for 100 ns with periodic boundary conditions on all axes [68,81,82]. Visualization of the evolution in time of the simulated complexes during the molecular dynamic simulation was obtained using VMD 1.9.4 [83].

The computer on which both the molecular docking and molecular dynamics studies were performed was equipped with an Intel Core 7700 K CPU and with a NVIDIA RTX 3060 GPU.

#### 3.9.7. Anti-Tryptic Activity (ATA)

This method is known also as an *in vitro* anti-arthritic activity. The analysis was performed according to the method of Oyedapo and Femurewa [55] with minor modification as described by Manolov et al. [66]. The reaction mixture contained 2 mL 0.06 mg/mL trypsin, 1 mL Tris–HCl buffer (20 mM, pH 7.4), and 1 mL test sample/standard (in methanol) of different concentrations (20–1000 μg/mL). The mixture was incubated at 37 °C for 5 min. Then, 1 mL of human albumin (4% *v/v*) was added. The mixture was incubated for an additional 20 min. To the mixture 2 mL of 70% perchloric acid was added for termination of the reaction. The cloudy suspension was cooled and centrifuged at 5000 rpm for 20 min. The absorbance of the supernatant was measured at 280 nm with a spectrophotometer (Camspec M508, Leeds, UK) against the control solution. The control solution was the sample/standard in methanol with different concentrations. Ibuprofen was used as the standard. The analysis was performed three times. The percentage of anti-tryptic activity (ATA) of the samples was evaluated by comparing with a blank sample. The blank sample was prepared as the test sample but with a small exception—perchloric acid was added before albumin.

(5) $$\%ATA=\left[\frac{A_{blank}-\left(A_{TS}-A_{CS}\right)}{A_{blank}}\right]\times 100$$

where *A_blank_* is the absorbance of the blank sample, *A_CS_* is the absorbance of the control solution (test sample in different concentrations), and *A_TS_* is the absorbance of the test samples.

### 3.10. In Vitro UV-B Photoprotection Study

The *in vitro* UV-B photoprotection assay of the individual fractions was performed using the spectrophotometric method reported by Mansur et al. (1986) [84]. A series of dilutions of 10, 5, 2.5, 1.25, 0.63, 0.31, and 0.078 mg/mL were prepared from a 20 mg/mL sample/standard. The absorbance of each of the diluted solutions was measured from 290 to 320 nm on a UV-VIS spectrophotometer (Camspec M508, Leeds, UK). Ethanol was used as a blank. Calculations were made according to Equation (6) and Table 5. Dibenzylideneacetone and cinnamic acid were used as standards. The assay was performed in triplicate.

(6) $$SPF=CF*\sum_{290}^{320}EE(\lambda )*I(\lambda )*Abs(\lambda ),$$

where *CF*—correction factor (equal to 10), *EE* (*λ*)*—*erythematous effect of wavelength radiation, *I* (*λ*)—sunlight intensity at the corresponding wavelength (*λ*), *Abs*(*λ*)—spectrophotometric reading of the absorption of the sample at the corresponding wavelength (*λ*).

### 3.11. Statistical Analysis

All of the analyses were performed in triplicate. The data were reported as mean ± SD. ANOVA data analysis was performed using the statistical tool SPSS 19.0 (SPSS Inc., Chicago, IL, USA) software. The level of significance was fixed at *p* < 0.05. The mean IC_50_ value was calculated from three replicates by interpolating the graphical dependency of activity on concentration.

## 4. Conclusions

In the present work, we investigated the polyphenolic composition of *H. italicum* fractionated with different polar solvents. We used the chromatographic methods HPLC-PDA and UHPLC-MS in negative and positive mode to analyze the fractions. Polyphenol separation is an efficient method by which polyphenols are separated by polarity. The phloroglucinols arzanol and its derivatives were identified in the hexane fraction. We found that the major components in the chloroform fraction were tremetones. Mono- and di-caffeoylquinic acids predominate in the polyphenol composition. The ethyl acetate fraction has a high yield, and a large part of the polyphenol composition is contained in it. In the ethyl acetate fraction, quercetin 3-*O*-glucuronide, quercetin acetyl-glycoside, isorhamnetin acetyl-glycoside, isorhamnetin caffeoyl-glycoside, quercetin caffeoyl-malonyl-glycoside, isorhamnetin coumaroyl-glycoside, coumaroyl-caffeoylquinic acid, and diCQA-acetyl-derivative*,* were identified, which have not been reported in the polyphenolic composition of *H. italicum* so far. Buthanol, as a more polar solvent than ethyl acetate, extracts the most polar components. It has the lowest content of polyphenols. Chromatographic analyses prove that it better extracts mono-caffeoylquinic acids. The analysis of the fractions shows that the polyphenolic complex is very heterogeneous. That is why we used standard compounds with a structure close to the polyphenolic composition, in order to determine which group of polyphenols would show higher *in vitro* biological activity. It also helps make the connection between structure and activity easier. In general, all fractions showed good antioxidant activity (HPSA, HRSA, MChA, and NOSA). The EtOAc fraction demonstrated higher antioxidant activity. This provides important information that caffeoylquinic acids and flavonoid glycosides are more active in the fight against oxidative and nitrosative stress. In addition, the same acids show significantly high UV-B protection, but this does not mean that nonpolar fractions should be neglected. It is phloroglucinols and tremetones that are characterized by the highest *in vitro* anti-inflammatory activity assessed as IAD. Phloroglucinols, tremetones, and caffeoylquinic acids showed higher ATA compared to standard ibuprofen. Despite the high yield of the buthanol fraction, it shows low activity. This is explained by the fact that it extracts a small number of polyphenolic components.

For this reason, fractionation has a great practical application. It not only divides the polyphenolic complex by polarity, but through it, pharmacologically active dosage forms can be prepared to find their medical application.

## Figures and Tables

**Table 1:** Total phenolic content (TPC), total tannin content (TTC), total flavonoid content (TFC), amount of quercetin flavonoids (QFs) determined by HPLC-PDA, amount of caffeoylquinic acids (CQAs) determined by HPLC-PDA, quantity of tremetones (TREs) determined by HPLC-PDA, quantity of phloroglucinols (PGHs) determined by HPLC-PDA.

Fractions	TPC	TTC	TFC	CQAs	QFs	TREs	PGHs
	*mgGAE/g Extr*	*mgTAE/g Extr*	*mgQE/g Extr*	*mg/g Extr*			
**n-H**	224.47 ± 31.00	170.64 ± 4.19	-	-	-	-	7.98 ± 0.26
**CHCl_3_**	194.00 ± 6.49	128.14 ± 9.52	-	-	-	26.84 ± 0.69	-
**EtOAc**	694.45 ± 17.08	468.35 ± 21.44	3.08 ± 0.09	45.82 ± 0.92	2.24 ± 0.05	-	-
**BuOH**	100.81 ± 6.28	57.80 ± 4.19	0.36 ± 0.01	12.68 ± 0.31	1.56 ± 0.04	-	-

**Table 2:** Hydrogen peroxide scavenging activity (HPSA), hydroxyl radical scavenging activity (HRSA), metal-chelating activity (MChA), nitric oxide scavenging activity (NOSA), inhibition of albumin denaturation (IAD), and anti-tryptic activity (ATA). Quercetin (Qrc), phloroglucinol (PGH), chlorogenic acid (ChA), and ibuprofen (Ibu) were used as standards.

Fractions	*HPSA*	*HRSA*	*NOSA*	*MChA*	*IAD*	*ATA*
	*IC*_50_ ± *SD*, μg/mL					
**n-H**	96.56 ± 2.66	94.58 ± 4.79	162.42 ± 13.16	8.87 ± 0.56	30.48 ± 2.33	102.93 ± 8.62
**CHCl_3_**	85.84 ± 1.08	123.65 ± 0.30	195.64 ± 19.03	6.27 ± 0.29	62.50 ± 1.69	108.92 ± 4.42
**EtOAc**	57.12 ± 1.14	92.23 ± 1.10	89.81 ± 2.09	5.60 ± 0.17	141.20 ± 3.27	84.19 ± 3.89
**BuOH**	218.86 ± 4.33	133.31 ± 5.66	236.71 ± 23.31	6.42 ± 0.24	892.21 ± 60.22	242.23 ± 44.98
**Standards**						
**Qrc**	69.25 ± 1.82	149.18 ± 9.27	64.82 ± 0.74	7.80 ± 0.17	-	-
**PGH**	52.98 ± 0.94	115.89 ± 4.85	98.11 ± 1.58	11.52 ± 1.55	-	-
**ChA**	56.70 ± 0.10	81.96 ± 2.80	75.37 ± 2.47	6.75 ± 0.66	-	-
**Ibu**	-	-	-	-	81.50 ± 4.95	259.82 ± 9.14

**Table 3:** The results of the molecular docking performed using AutoDock vina expressed as the variation of Gibbs free energy (ΔG kcal/mol).

Compound	Sudlow 1	Sudlow 2	Site 3	Cleft
**arzanol**	−7.9	**−9.6**	−9.0	−7.0
**(*R*)-bitalin A**	−6.5	−8.0	**−8.4**	−7.4
**(*S*)-bitalin A**	−6.6	−8.1	**−8.8**	−7.5

**Table 4:** The average root-mean-square deviation of the backbone of the albumin (RMSD-protein), the average root-mean-square deviation of the heavy atoms of the ligands (RMSD-ligand), the average of the total radius of gyration of the albumin (Rg), and the average number of hydrogen bonds (NoHB) between the docked ligands and the albumin in the systems evaluated for 100 ns in the in silico molecular dynamics study.

System Evaluated	RMSD-Protein (nm)	RMSD-Ligand (nm)	RG (nm)	NoHB (no/ns)
arzanol in site Sudlow 2	0.32	0.36	2.78	0.11
(*R*)-bitalin A in site 3	0.31	0.48	2.74	0.01
(*S*)-bitalin A in site 3	0.28	0.24	2.76	0.33
apo albumin	0.38	N/A	2.78	N/A

**Table 5:** Relationship between erythematous effect and radiation intensity at each wavelength.

*λ*, nm	*EE* (*λ*) × *I* (*λ*)
290	0.015
295	0.0817
300	0.2874
305	0.3278
310	0.1864
315	0.0839
320	0.018
∑	1.0

## Data Availability

The data presented in this study are available in this article and in the supporting Supplementary Material.

## Supplementary Materials

The following supporting information can be downloaded at: https://www.mdpi.com/article/10.3390/molecules28176198/s1. Figure S1. Chromatographic profile of the fractionated polyphenolic complex from *H. italicum* obtained by HPLC-PDA. (A) Chromatographic profile of EtOAc fraction. The major components in the EtOAc fraction were caffeoylquinic acids, while quercetin, isorhamnetin, and kaempferol glycosides were present in low amounts. (B) Chromatographic profile of BuOH fraction. In the BuOH fraction, the presence of mono-caffeoylquinic acids was established by UV spectra; Figure S2. Chromatographic profile of the fractionated polyphenolic complex from *H. italicum* obtained by HPLC-PDA. (A) Chromatographic profile of hexane fraction. The main components in the hexane fraction are phloroglucinols. (B) Chromatographic profile of CHCl3 fraction. The main components in the CHCl_3_ fraction are tremetones; Figure S3. Mass spectrum of methylarzanol obtained by positive ion ESI-MS/MS; Figure S4. Proposed fragmentation of protonated methylarzanol [M+H]^+^; Figure S5. Mass spectrum of arzanol derivative obtained by positive ion ESI-MS/MS; Figure S6. Proposed fragmentation of protonated arzanol derivative [M+H]^+^; Figure S7. Mass spectrum of heliarzanol obtained by positive ion ESI-MS/MS; Figure S8. Proposed fragmentation of protonated heliarzanol [M+H]^+^; Figure S9. Mass spectrum of helipyron obtained by positive ion ESI-MS/MS; Figure S10. Proposed fragmentation of protonated helipyron [M+H]^+^; Figure S11. Mass spectrum of italipyron obtained by positive ion ESI-MS/MS; Figure S12. Proposed fragmentation of protonated italipyron [M+H]^+^; Figure S13. Mass spectrum of gnaphaliol obtained by positive ion ESI-MS/MS; Figure S14. Proposed fragmentation of protonated gnaphaliol [M+H]^+^; Figure S15. Mass spectrum of 3-actoxy-10-hydroxytremeton obtained by positive ion ESI-MS/MS; Figure S16. Proposed fragmentation of protonated 3-actoxy-10-hydroxytremeton [M+H]^+^; Figure S17. Mass spectrum of 13-(2-methylpropanoyloxy)toxol obtained by positive ion ESI-MS/MS; Figure S18. Proposed fragmentation of protonated 13-(2-methylpropanoyloxy)toxol [M+H]^+^; Figure S19. Mass spectrum of 3-hydroxy-10-propanoyloxy tremeton obtained by positive ion ESI-MS/MS; Figure S20. Proposed fragmentation of protonated 3-hydroxy-10-propanoyloxy tremeton [M+H]^+^; Figure S21. RMSD of protein backbone when complexed with arzanol, (*R*)-bitalin A, and (*S*)-bitalin A compared with the apo form; Figure S22. RMSD of arzanol, (*R*)-bitalin A, and (*S*)-bitalin A when complexed with albumin; Figure S23. Radius of gyration of protein atoms when complexed with arzanol, (*R*)-bitalin A, and (*S*)-bitalin A compared with the apo form; Figure S24. Number of hydrogen bonds between albumin and arzanol, (*R*)-bitalin A, and (*S*)-bitalin A.

## Abbreviations

HPLC-PDA	High-performance liquid chromatography PDA detector
UHPLC-MS/MS	Ultra-high-performance liquid chromatography-MS/MS
CQA	Caffeoylquinic acid
HPSA	Hydrogen peroxide scavenging activity
HRSA	Hydroxyl radical scavenging activity
MChA	Metal-chelating activity
NOSA	Nitric oxide scavenging activity
IAD	Inhibition albumin denaturation
ATA	Antitryptic activity
TPC	Total phenolic content
TTC	Total tannin content
TFC	Total flavonoid content
QFs	Quercetin flavonoids
PGH	Phloroglucinol
ChA	Chlorogenic acid
TRE	Tremeton
SPF	Sun protection factor
DiBA	Dibenzylideneacetone
CA	Cinnamic acid

## Appendix A

**Table A1:** Peak number, RT, molecular ion, and fragment characteristic ions (UHPLC-MS/MS) of polyphenolic components identified in hexane, chloroform, ethyl acetate, and butanol fractions.

Fractions	Peak №	Compound Name	RT	UHPLC-MS						Ref.
				[M-H]^−^	∆m, ppm	MS/MS	[M+H]^+^	∆m, ppm	MS/MS	
**n-H Fraction**	1	Arzanol	21.27	401.1607	0.25	401, 247, 235, 205, 191, 166, 153, 123, 109	403.1750	−0.25	403, 347, 249, 237, 193, 181, 175, 167, 163, 155, 139	[8,35,38,85,86]
	2	Helipyrone	21.81	319.1193	1.87	153, 109	321.1333	0.00	321, 167, 155, 139, 57	[85,86]
	3	3-methyl-arzanol	22.51	415.1767	1.20	415, 247, 235, 191, 167	417.1910	0.48	417, 361, 249, 237, 193, 181, 175, 169, 163	[35,85,86]
	4	Italipyrone	22.77	399.1455	1.55	399, 233, 189, 153, 147, 109	401.1598	0.75	401, 359, 247, 235, 229, 167, 155, 139	[85,86]
	5	Methylarzanol-isomer	23.34	415.1767	1.20	415, 261, 249, 205, 153, 137, 109	417.1910	0.48	417, 361, 263, 251, 207, 195, 189, 177, 167, 155, 139	[35,86]
	6	Heliarzanol	23.52	445.1879	2.47	291, 279, 210, 195, 168, 153, 125, 109	447.2002	−2.46	447, 429, 321, 293, 281, 225, 207, 195, 213, 177, 167, 155, 139	[8,85,86]
	7	Arzanol-derivative	24.07	429.1922	0.70	429, 247, 235, 191, 166, 137	431.2065	0.23	431, 375, 249, 237, 193, 181, 175, 163	[8]
	8	Arzanol-derivative	24.97	429.1921	0.47	429, 275, 263, 249, 219, 205, 167, 161, 153	431.2066	0.23	431, 375, 247, 265, 251, 221, 209, 203, 195, 191, 181, 167, 163, 155, 149, 139	[8]
	9	Italipyron-isomer	25.61	399.1465	1.50	399, 233, 189, 153, 147, 109	401.1594	−0.25	401, 247, 235, 229, 217, 193, 167, 155, 147, 139	[85]
	10	Arzanol-isomer	25.76	401.1635	7.23	247, 235, 179, 163, 153, 137, 109	403.1752	0.25	403, 347, 249, 237, 193, 181, 175, 167, 163, 155	
**CHCl_3_ Fraction**	11	Caffeic acid	6.52	179.0343	−3.91	179, 164, 135, 117, 108	181.0491	−2.21	181, 163, 153, 137, 135, 125	[11,12,13,14,85]
	12	Gnaphaliol	7.56	-	-	-	235.0959	−2.55	235, 217, 199, 189, 175, 171, 163, 157, 149, 147, 145, 129, 121	[5,11]
	13	Bitalin A	11.17	-	-	-	219.1014	−0.91	201, 183, 173, 165, 159, 157, 155, 149, 144, 141, 131	[5,6,11,12]
	14	Methoxyluteolin	11.86	315.0512	0.63	300, 271, 255, 243, 227, 199, 165, 139, 137	317.0647	−2.84	317, 302, 274, 245, 228, 187, 168, 125, 121, 107	[14,19]
	15	Italipyrone-isomer	12.04	-	-	-	401.1585	−2.49	401, 247, 235, 187, 167	[85]
	16	Quercetin-3-methyl ether	12.12	315.0518	2.54	300, 271, 255, 243, 227, 199, 164, 151	317.0658	0.63	317, 302, 299, 274, 228, 153, 137, 121	[19,85]
	17	3-acetoxy-10-hydroxytremeton	12.14	-	-	-	277.1064	−2.53	219, 217, 201, 199, 175, 157, 147, 133, 129	[5]
	18	13-(2-methylpropanoyloxy)toxol	12.75	-	-	-	305.1385	0.33	235, 217, 201, 189, 183, 179, 168, 165, 159, 157, 155, 151, 141, 131, 127, 123	[5]
	19	Luteolin	12.85	285.0405	0.00	151, 133, 107	-	−	-	[20,85]
	20	Naringenin		271.0613	0.37	271, 253, 215, 151, 137, 125, 107	-	−	-	[14,20,85]
	21	Herbacetin-3-methyl ether	13.09	315.0514	1.27	300, 271, 255, 243, 227, 164, 163, 151, 124, 107	317.0648	−2.52	317, 302, 285, 274, 245, 229, 217, 181, 165, 153, 121, 137	[19,85]
	22	3-hydroxy-10-propionyloxytremeton	13.43	-	-	-	291.1223	−1.37	291, 273, 235, 223, 217, 205, 189, 179, 167, 155, 151, 139, 137, 123, 113	[5]
	23	Kaempferol-3-methyl ether	13.95	299.0562	0.33	299, 284, 255, 239, 227, 211, 199, 183, 139	301.0707	0.00	301, 286, 285, 257, 240, 212, 187, 171, 107	[19,85]
	24	Pinocembrin	16.76	255.0662	−0.39	227, 213, 185, 171, 151, 145, 107	257.0801	−2.72	257, 153, 131, 123, 107, 103	[6,19,85]
	25	Galangin	17.00	269.0456	0.37		271.0601	0.00	253, 242, 215, 197, 165, 153, 141, 131	[19,85]
	26	Galangin-3-methyl ether	17.73	283.0613	0.35	268, 239, 211, 195, 167	285.0759	0.70	271, 269, 242, 213, 137	[19,85]
**EtOAc Fraction**	27	Neochlorogenic acid	2.18	353.0886	2.27	191, 179, 161, 135	-	−	-	[13,85]
	28	Chlorogenic acid	3.71	353.0880	0.57	191, 173, 161	355.1021	−0.84	163, 154, 135, 117	[11,12,14,85]
	29	Criptochlorogenic acid	4.20	353.0885	1.98	191, 179, 173, 135	355.1021	−0.84	355, 285, 268, 163, 145, 135, 117	[13,85]
	30	5-*O*-Feruloylquinic acid	6.25	367.1041	1.63	367, 191, 179, 161, 135	369.1182	0.54	163, 145, 135, 117	[85,86]
	31	Myricetin-glycoside	6.76	479.0837	1.25	479, 317, 165, 139	481.0970	−1.46	481, 319, 169, 137	[14,85]
	32	Feruloylquinic acid-isomer	7.25	367.1041	1.63	367, 191, 179, 161, 135	369.1182	0.54	163, 145, 135, 117	[85]
	33	Quercetin-3-*O*-glucuronide	7.94	477.0686	2.31	477, 301, 273, 255, 227, 211, 179, 151, 121, 107	479.0521	0.21	479, 303, 285, 257, 229, 201, 165, 153, 137, 109	
	34	Quercetin-glycoside	8.15	463.0889	1.51	463, 300, 271, 255, 243, 227, 179, 151, 107	465.1023	−1.08	465, 303, 285, 229, 153, 137	[11,85]
	35	3.4-diCaffeoylquinic acid	9.59	515.1196	0.19	515, 353, 335, 191, 179, 173, 161, 135	517.1337	−0.77	163, 145, 135, 117	[11,85]
	36	3.5-diCaffeoylquinic acid	9.81	515.1195	0	353, 191, 179, 161, 135	517.1334	−1.35	499, 163, 145, 135, 117	[11,85]
	37	1.5-diCaffeoylquinic acid	10.06	515.1199	0.78	353, 335, 191, 179, 173, 161, 135	517.1339	−0.39	499, 163, 145, 135, 117	[13,85]
	38	Isorhamnetin-glycoside	10.16	477.1044	1.26	477, 315, 314, 299, 285, 271, 257, 243, 227, 199, 179, 151	479.1181	−2.71	479, 317, 302, 274, 229, 181, 153, 121, 109	[11,85,86]
	39	4.5-diCaffeoylquinic acid	10.49	515.1197	0.39	515, 353, 191, 179, 173, 161, 135, 93	517.1335	−1.16	499, 163, 145, 135, 117	[11,12,85]
	40	Quercetin-glycoside	10.72	463.0890	1.73	463, 301, 273, 257, 229	465.1026	−0.43	465, 303, 285. 257, 229, 153, 137, 127, 121	[11,85]
	41	3-Feruloyl-5-caffeoylquinic acid	11.08	529.1362	2.08	529, 367, 193, 173, 161, 134, 117, 93	-	-	-	[85]
	42	Quercetin-acetylglycoside	11.25	505.1003	2.97	505, 301, 285, 273, 179, 151, 137, 121, 107	-	-	-	
	43	Isorhamnetin-acetylglycoside	11.29	519.1152	1.54	314, 299, 285, 271, 271, 257, 243, 227	-	-	-	
	44	3-Feruloyl-5-caffeoylquinic acid isomer	11.27	529.1362	2.08	367, 193, 191, 179, 173, 161, 135, 93	-	-	-	[85]
	45	Coumaroyl-Caffeoylquinic acid	11.28	499.1265	3.81	499, 353, 191, 179, 173, 161, 135	-	-	-	
	46	Quercetin coumaroyl-glycoside	11.35	609.1271	3.45	609, 463, 300, 271, 255, 243, 227, 179, 151, 107	611.1393	−0.33	449. 303, 165, 147, 119	[85,86]
	47	4-Feruloyl-5-caffeoylquinic acid	11.47	529.1364	2.64	529, 367, 353, 349, 193, 191, 179, 173, 161, 135, 133, 117	-	-	-	[85]
	48	Isorhamnetin-caffeoylglycoside	11.49	639.1373	2.82	639, 477, 315, 300, 271, 255, 243, 227, 151, 135, 107	-	-	-	
	49	Quercetin-coumaroyl-glycoside-isomer	11.55	609.1270	3.28	609, 463, 300, 271, 255, 243, 227, 179, 151, 107	611.1398	0.49	611, 303, 147, 163	[85,86]
	50	Quercetin	11.75	301.0357	1.00	301, 179, 151, 121, 107	303.0490	−2.97	303, 285, 257, 229, 201, 153, 137, 121,	[85]
	51	Quercetin-caffeoyl-malonylglycoside	11.77	711.1224	2.95	667, 505, 487, 365, 300, 271, 255, 243, 227, 179, 151, 107	713.1341	−0.95	713, 411, 303	
	52	Tiliroside	11.90	593.1314	2.19	593, 447, 385, 285, 255, 227, 211, 151, 132, 117	-	-	-	[6,11,85,86]
	53	Isorhamnetin-coumaroyl-glycoside	12.05	623.1420	2.25	623, 508, 315, 299, 271, 243, 182, 145, 135, 133	-	-	-	
	54	diCQA-derivative (acyl)	12.55	681.1848		639, 515, 353, 191, 179, 173, 161, 135, 93	-	-	-	
	55	Coumaroylquinic acid	20.05	337.0937	2.37	337, 277, 163, 119	-	-	-	[85]
**BuOH Fraction**	56	Hydroxyphtalid-glycoside	2.02	327.0725	0.92	327, 207, 165, 121	-	-	-	[85]
	57	Caffeic acid-glycoside 1	3.65	341.0885	2.05	341, 179, 135	-	-	-	[85]
	58	Caffeic acid-glycoside 2	4.03	387.0937	1.29	179, 151, 135	-	-	-	[85]
	59	Caffeoylquinic acid-isomer	5.34	353.0885	1.98	191, 173, 161	-	-	-	[85]
	60	Quercetin diglycoside	6.63	625.1432	3.52	463, 301, 271, 255, 243, 179, 151, 107	-	-	-	[85]
