# Supplementary material for: HPLC Analysis and In Vitro and In Silico Evaluation of the Biological Activity of Polyphenolic Components Separated with Solvents of Various Polarities from Helichrysum italicum
Source: Molecules. 2023 Aug 23;28(17):6198. doi: 10.3390/molecules28176198 (PMC10488648; doi:10.3390/molecules28176198)
Supplement: Supplementary file 1 [file molecules-28-06198-s001.zip › molecules-2561932-supplementary.pdf]

Supplementary Materials:

HPLC Analysis and In Vitro and In Silico Evaluation of the Biological Activity of Polyphenolic Components Separated with Solvents of Various Polarities from *Helichrysum italicum*

Dimitar Bojilov *, Stanimir Manolov *, Sezan Ahmed, Soleya Dagnon, Iliyan Ivanov, Gabriel Marc, Smaranda Oniga, Ovidiu Oniga, Paraskev Nedialkov and Silviya Mollova

Contents

Figure S1. Chromatographic profile of the fractionated polyphenolic complex from *H. italicum* obtained by HPLC-PDA. (A) Chromatographic profile of EtOAc fraction. The major components in the EtOAc fraction were caffeoylquinic acids, while quercetin, isorhamnetin, and kaempferol glycosides were present in low amounts. (B) Chromatographic profile of BuOH fraction. In the BuOH fraction, the presence of mono caffeoylquinic acids was established by UV spectra. 3

Figure S2. Chromatographic profile of the fractionated polyphenolic complex from *H. italicum* obtained by HPLC-PDA. (A) Chromatographic profile of hexane fraction. The main components in the hexane fraction are phloroglucinols (B) Chromatographic profile of CHCl3 fraction. The main components in the CHCl3 fraction are tremetones.......... 4

Figure S3. Mass spectrum of methylarzanol obtained by positive ion ESI-MS/MS 5

Figure S4. Proposed fragmentation of protonated methylarzanol $[M+H]^+$ 5

Figure S5. Mass spectrum of arzanol derivative obtained by positive ion ESI-MS/MS 6

Figure S6. Proposed fragmentation of protonated arzanol derivative $[M+H]^+$. 6

Figure S7. Mass spectrum of heliarzanol obtained by positive ion ESI-MS/MS 7

Figure S8. Proposed fragmentation of protonated heliarzanol $[M+H]^+$. 8

Figure S9. Mass spectrum of helipyron obtained by positive ion ESI-MS/MS 9

Figure S10. Proposed fragmentation of protonated helipyron $[M+H]^+$.......... 9

Figure S11. Mass spectrum of italipyron obtained by positive ion ESI-MS/MS 10

Figure S12. Proposed fragmentation of protonated italipyron $[M+H]^+$.......... 10

Figure S13. Mass spectrum of gnaphaliol obtained by positive ion ESI-MS/MS.......... 11

Figure S14. Proposed fragmentation of protonated gnaphaliol $[M+H]^+$. 11

Figure S15. Mass spectrum of 3-actoxy-10-hydroxytremeton obtained by positive ion ESI-MS/MS. 12

Figure S16. Proposed fragmentation of protonated 3-actoxy-10-hydroxytremeton $[M+H]^+$. 12

Figure S17. Mass spectrum of 13-(2-methylpropanoyloxy)toxol obtained by positive ion ESI-MS/MS 13

Figure S18. Proposed fragmentation of protonated 13-(2-methylpropanoyloxy)toxol $[M+H]^+$. 13

Figure S19. Mass spectrum of 3-hydroxy-10-propanoyloxy tremeton obtained by positive ion ESI-MS/MS 14

Figure S20. Proposed fragmentation of protonated 3-hydroxy-10-propanoyloxy tremeton $[M+H]^+$... 14

Figure S21. RMSD of protein backbone when complexed with arzanol, (R)-bitalin A and (S)-bitalin A compared with the and apo form 15

Figure S22. RMSD of arzanol, (R)-bitalin A and (S)-bitalin A when complexed with albumin 15

Figure S23. Radius of gyration of protein atoms when complexed with arzanol, (R)-bitalin A and (S)-bitalin A compared with the and apo form 15

Figure S24. Number of hydrogen bonds between albumin and arzanol, (R)-bitalin A and (S)-bitalin A........15

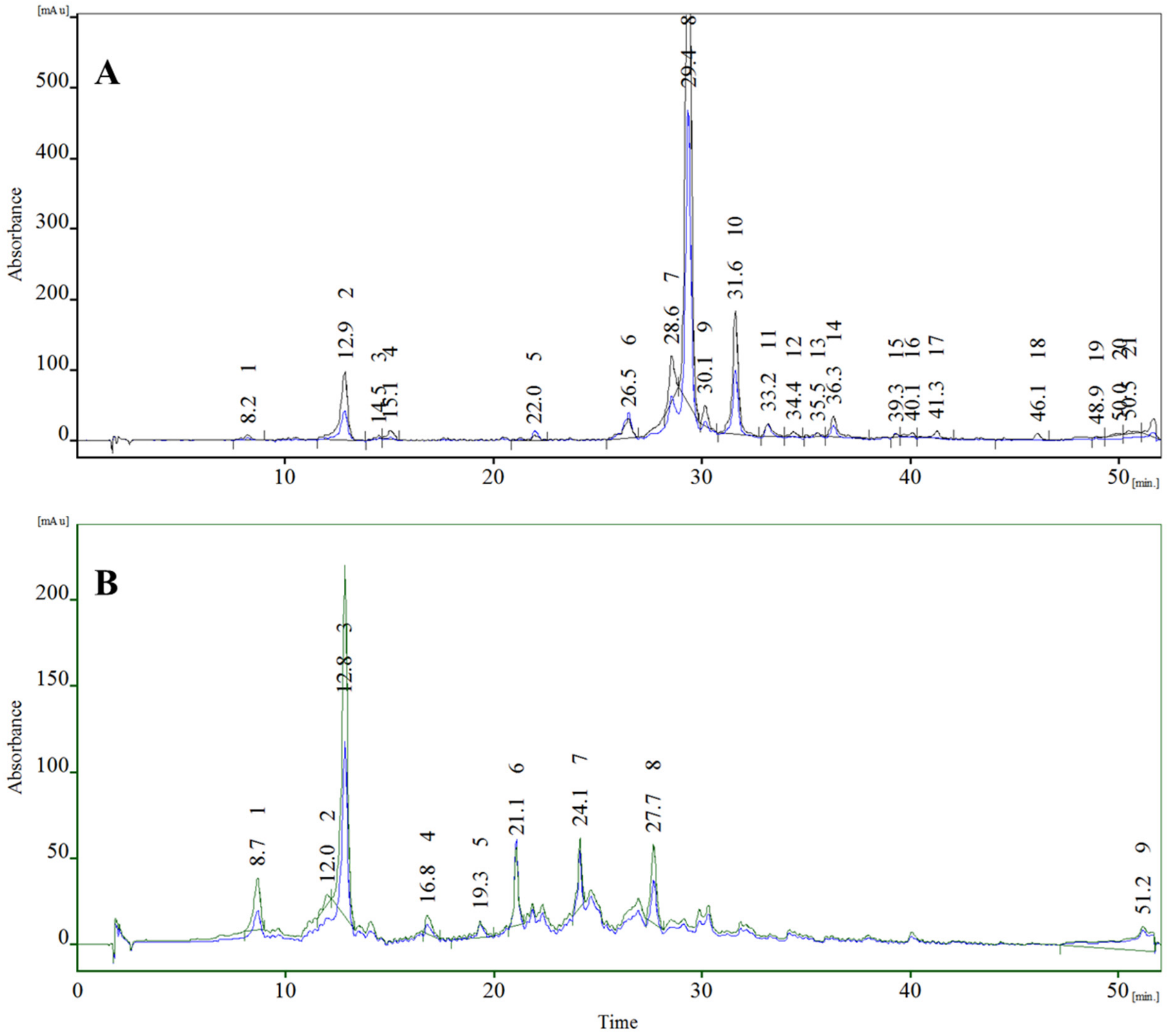

Figure S1. Chromatographic profile of the fractionated polyphenolic complex from *H. italicum* obtained by HPLC-PDA. (A) Chromatographic profile of EtOAc fraction. The major components in the EtOAc fraction were caffeoylquinic acids, while quercetin, isorhamnetin, and kaempferol glycosides were present in low amounts. (B) Chromatographic profile of BuOH fraction. In the BuOH fraction, the presence of mono caffeoylquinic acids was established by UV spectra.

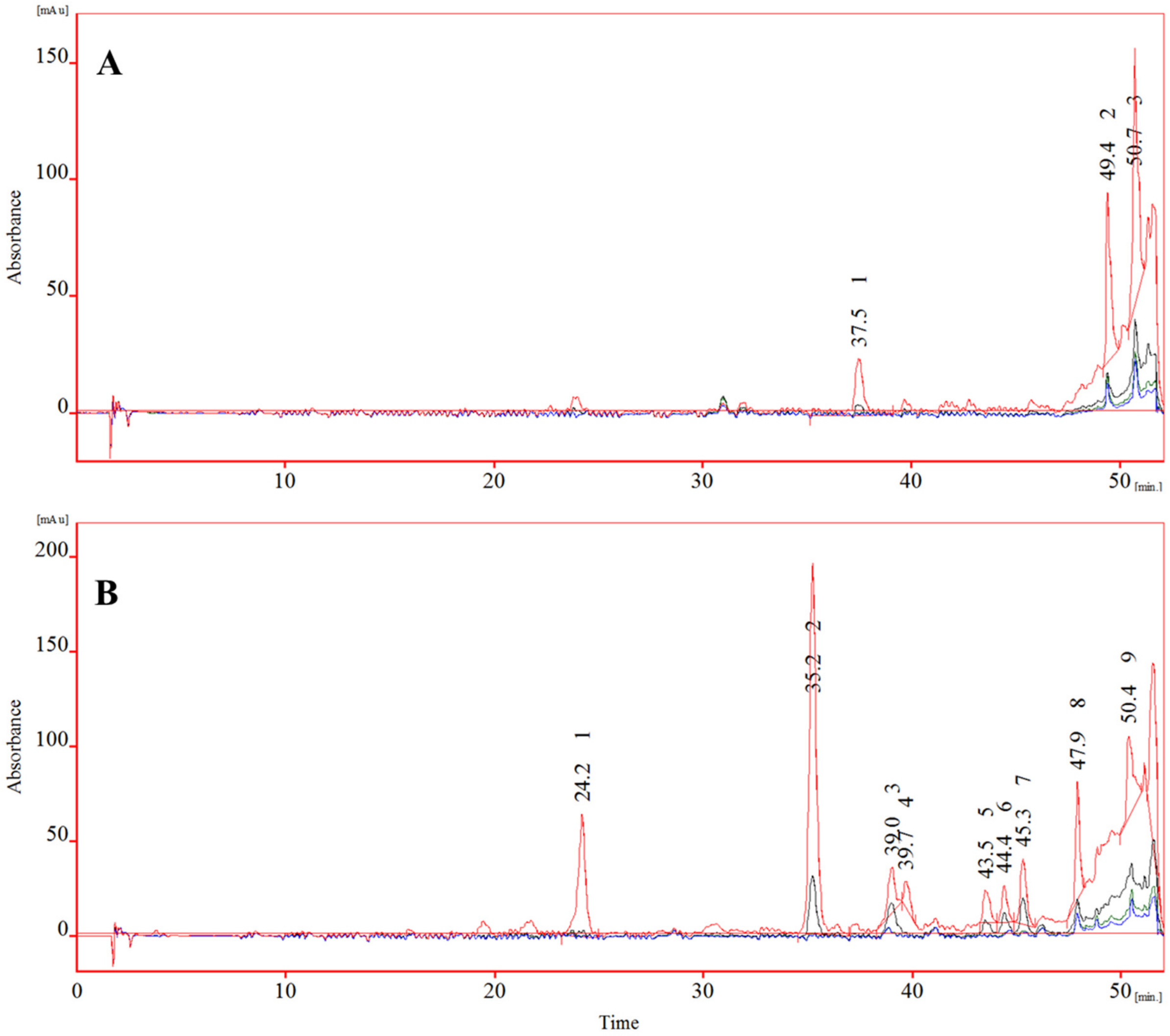

Figure S2. Chromatographic profile of the fractionated polyphenolic complex from *H. italicum* obtained by HPLC-PDA. (A) Chromatographic profile of hexane fraction. The main components in the hexane fraction are phloroglucinols (B) Chromatographic profile of CHCl3 fraction. The main components in the CHCl3 fraction are tremetones.

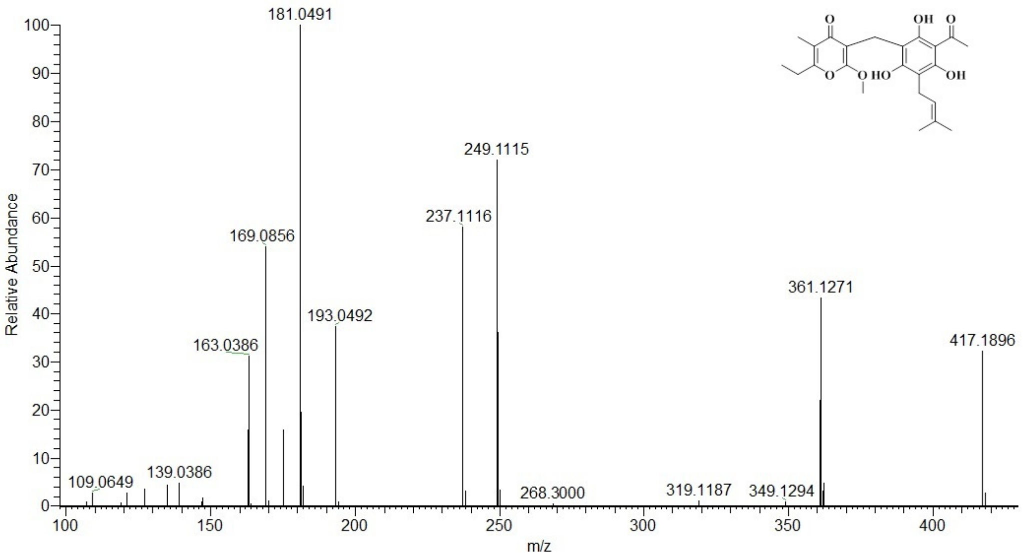

Figure S3. Mass spectrum of methylarzanol obtained by positive ion ESI-MS/MS

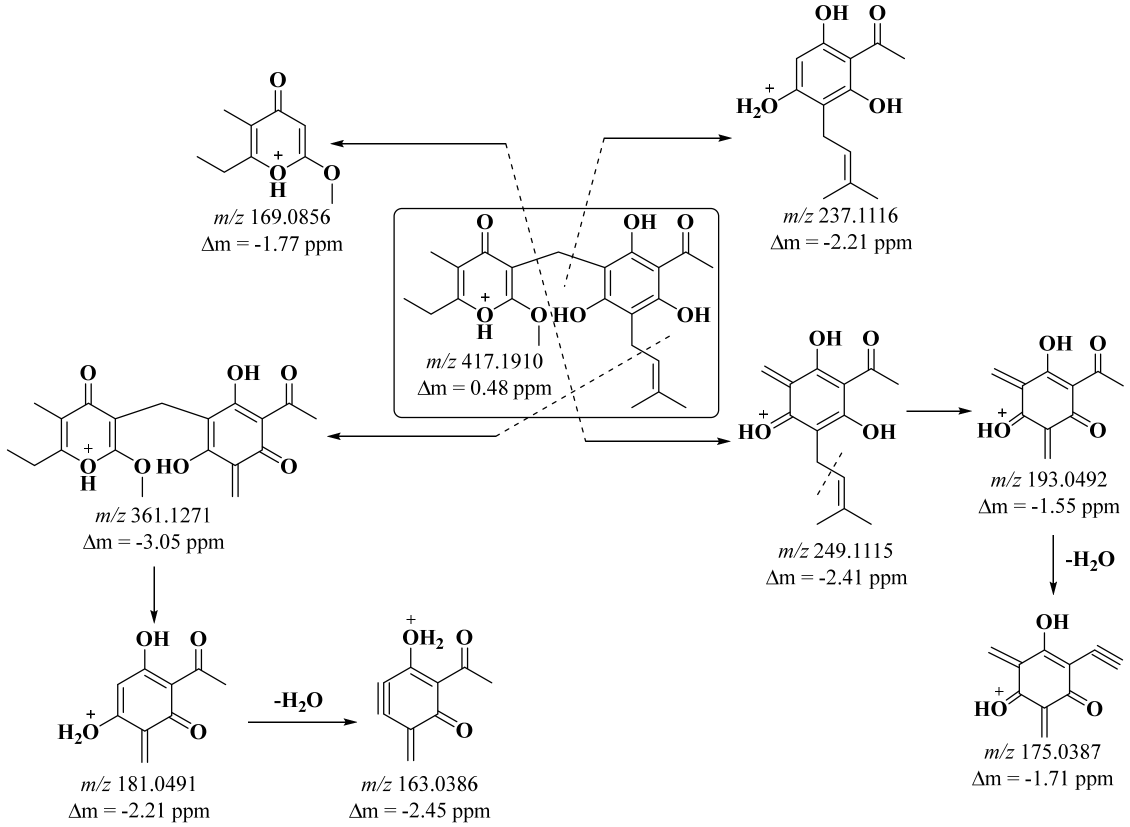

Figure S4. Proposed fragmentation of protonated methylarzanol $[M+H]^+$.

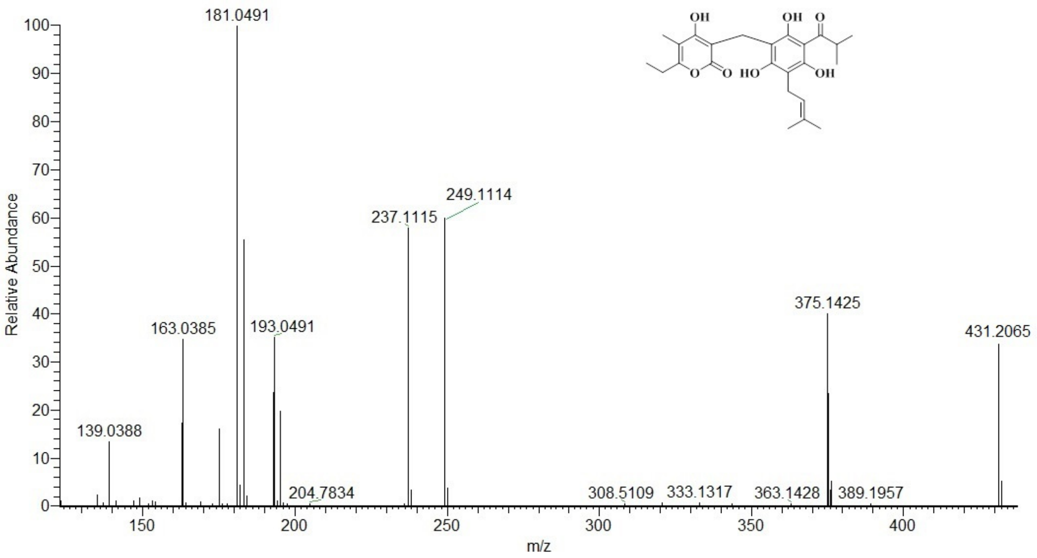

Figure S5. Mass spectrum of arzanol derivative obtained by positive ion ESI-MS/MS

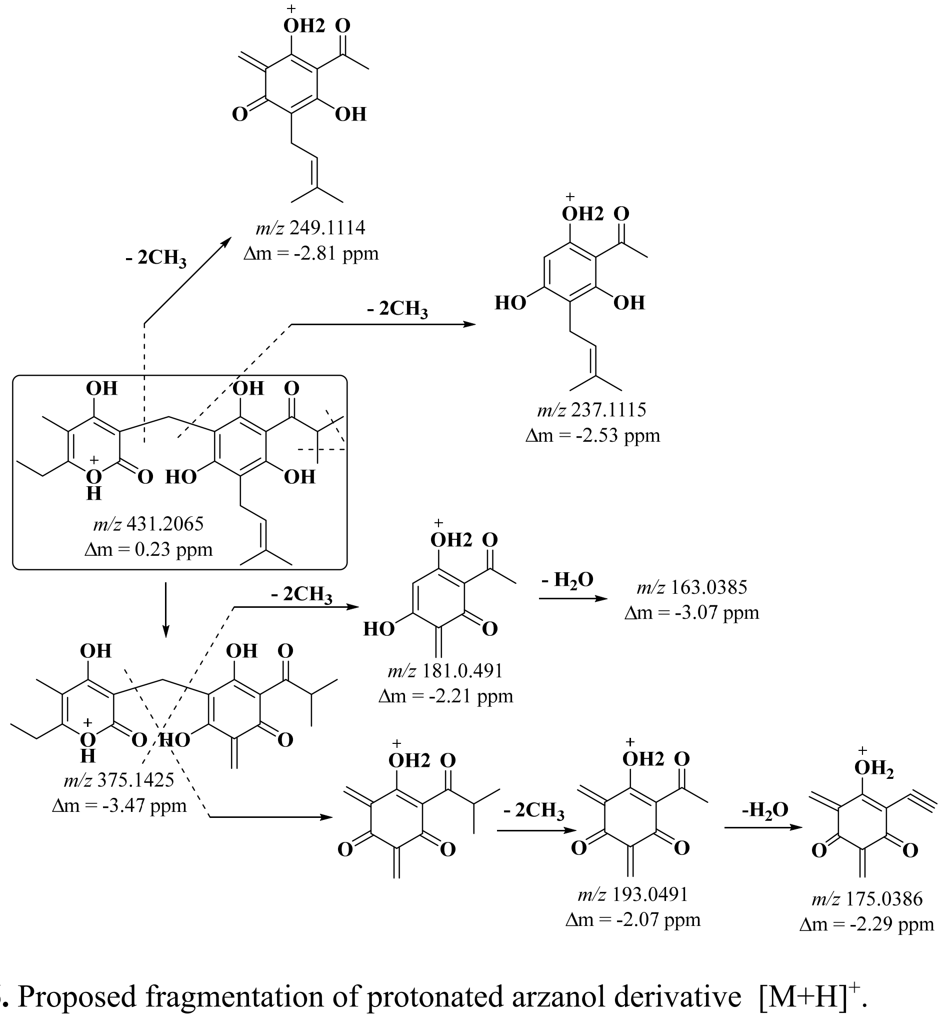

Figure S6. Proposed fragmentation of protonated arzanol derivative $[M+H]^+$.

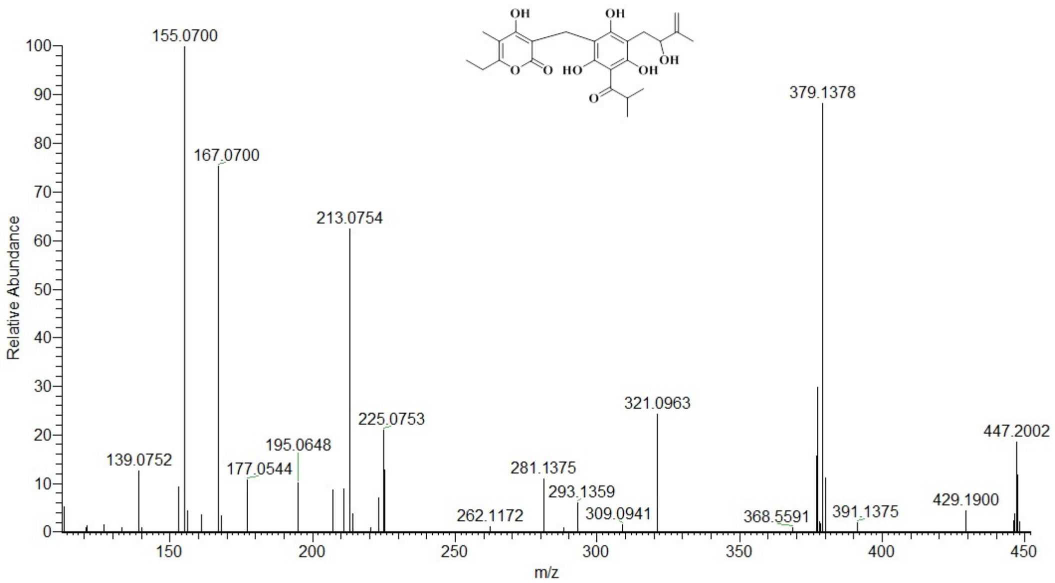

Figure S7. Mass spectrum of heliarzanol obtained by positive ion ESI-MS/MS

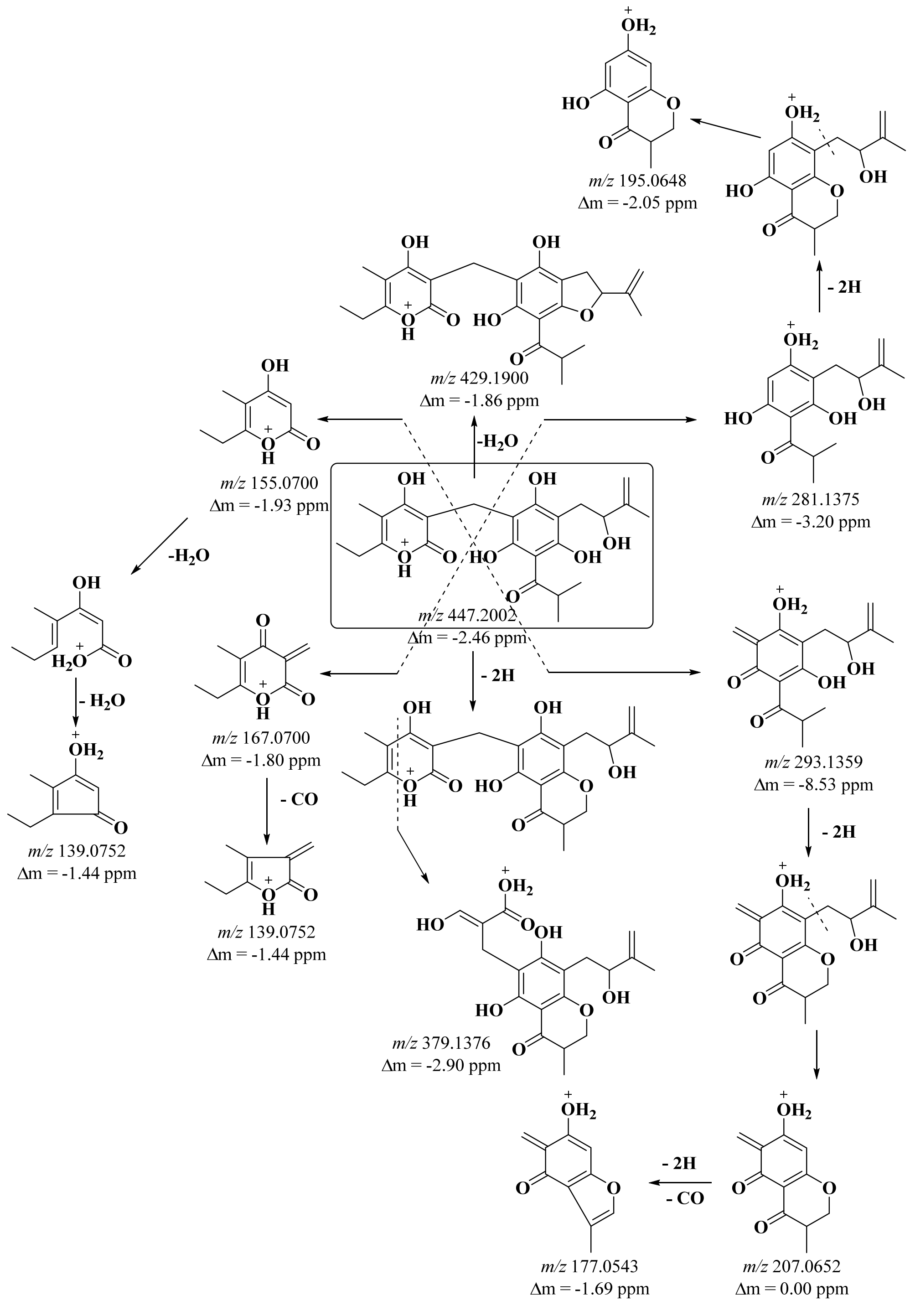

Figure S8. Proposed fragmentation of protonated heliarzanol $[M+H]^+$.

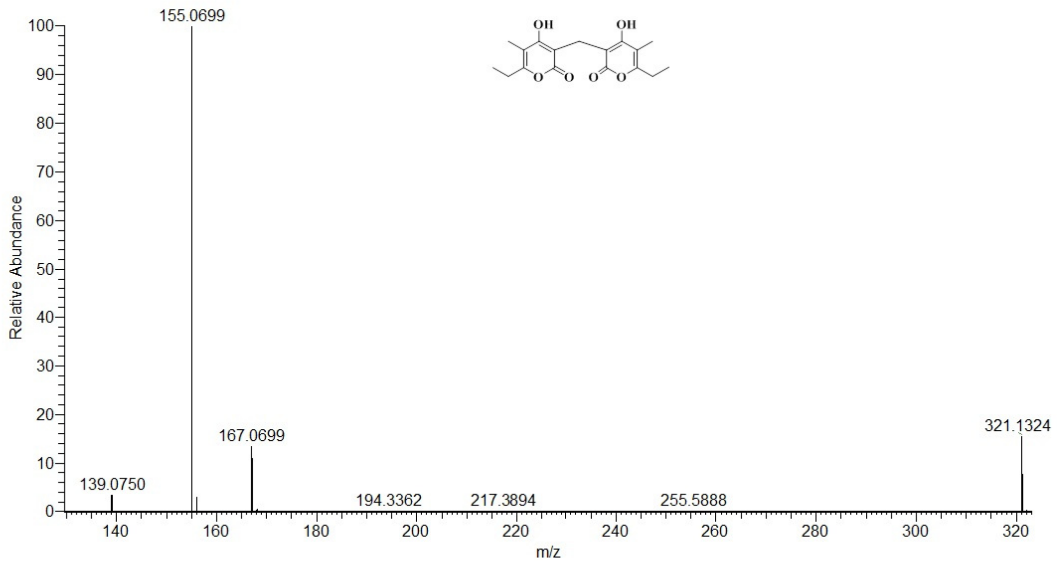

Figure S9. Mass spectrum of helipyron obtained by positive ion ESI-MS/MS

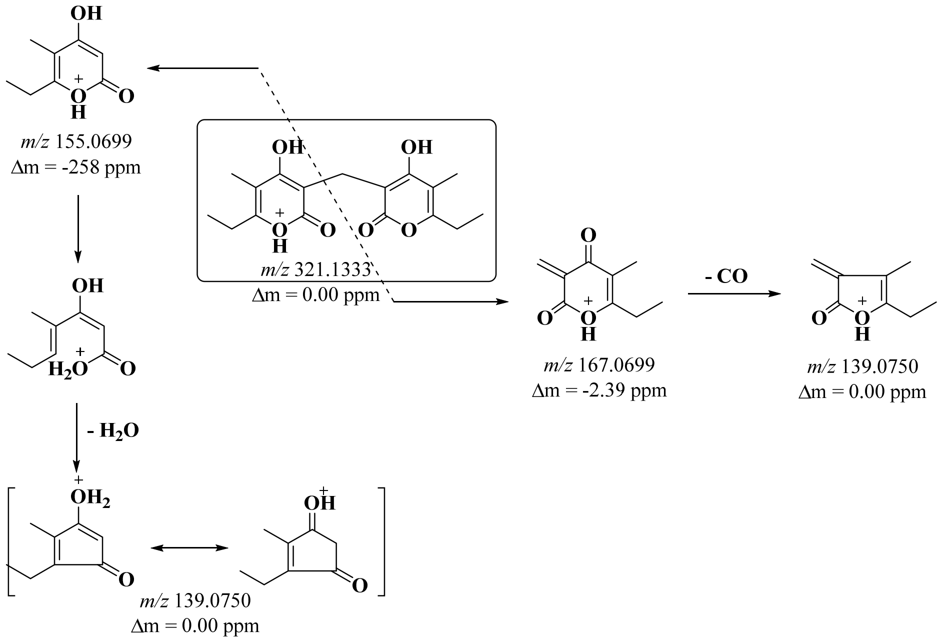

Figure S10. Proposed fragmentation of protonated helipyron $[M+H]^+$.

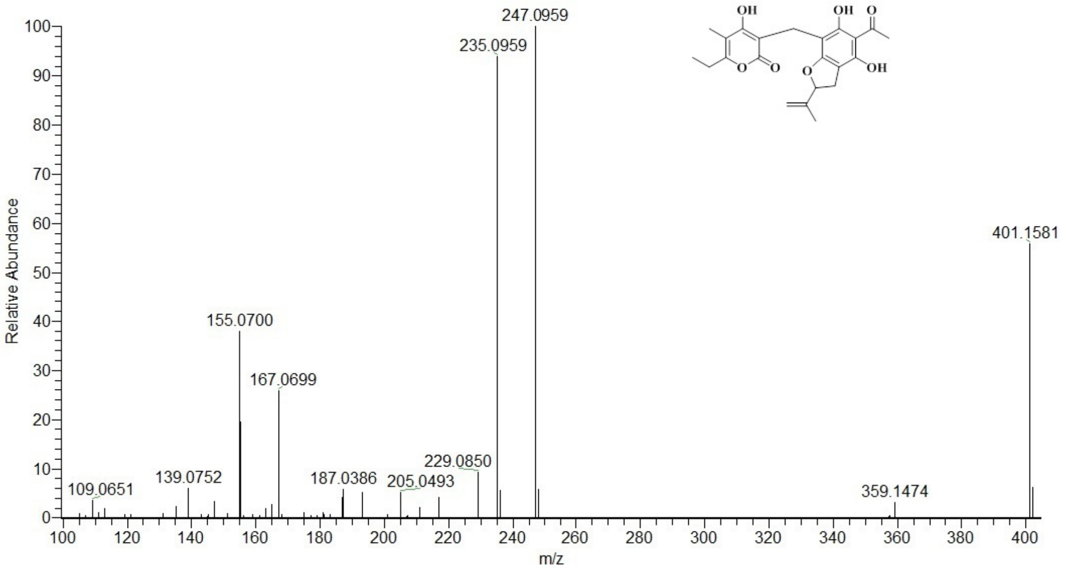

Figure S11. Mass spectrum of italipyron obtained by positive ion ESI-MS/MS

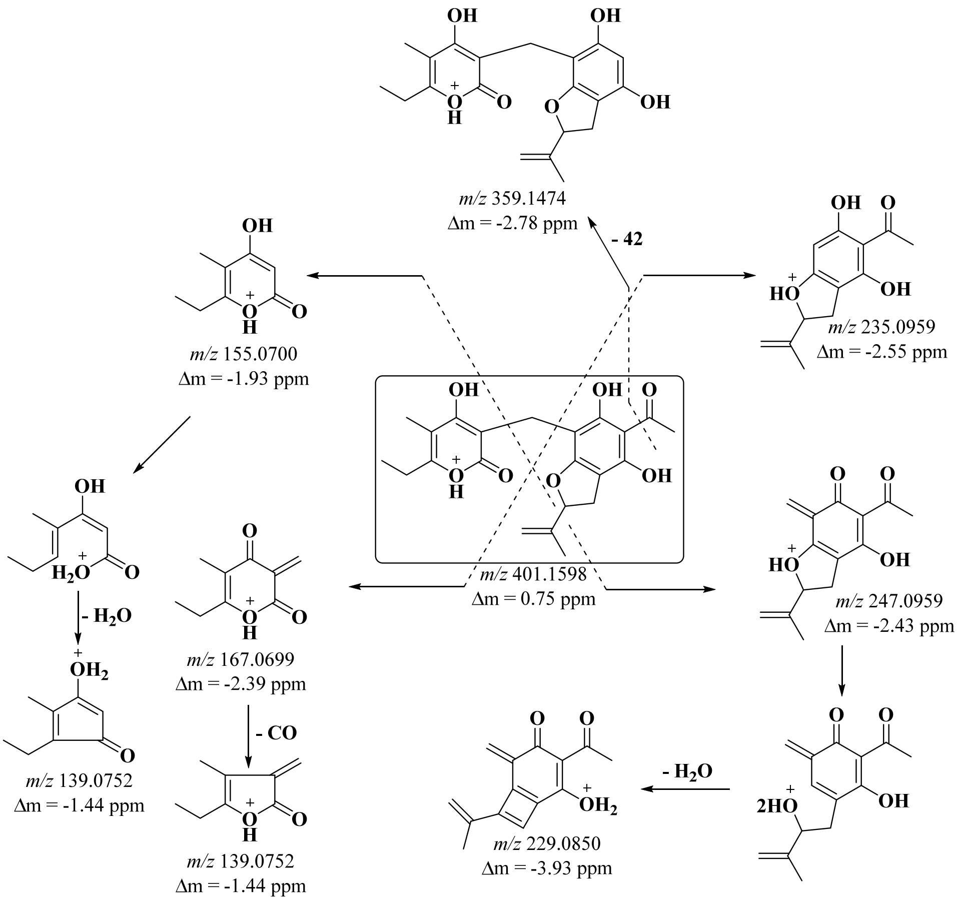

Figure S12. Proposed fragmentation of protonated italipyron $[M+H]^+$.

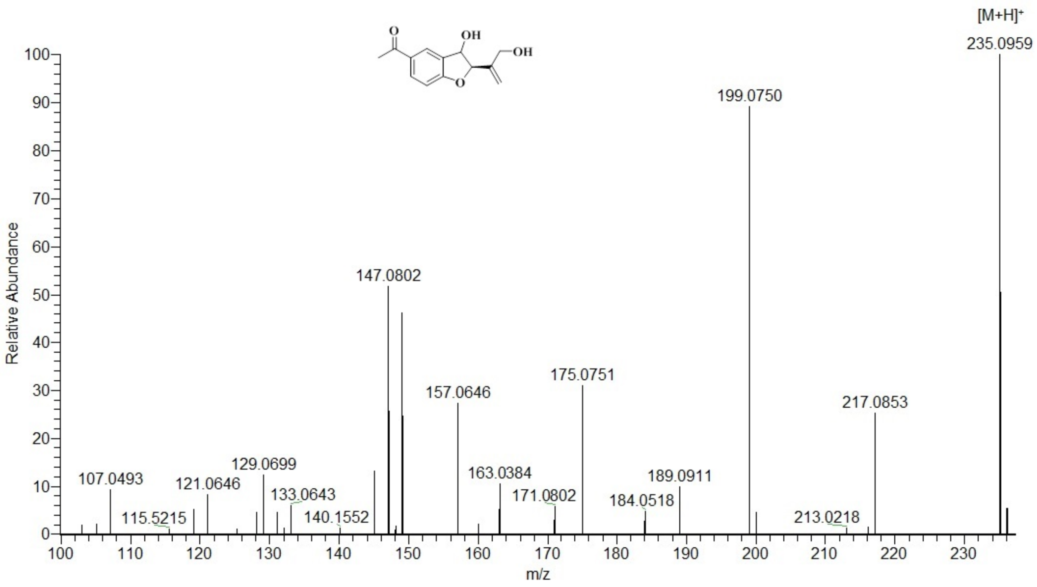

Figure S13. Mass spectrum of gnaphaliol obtained by positive ion ESI-MS/MS

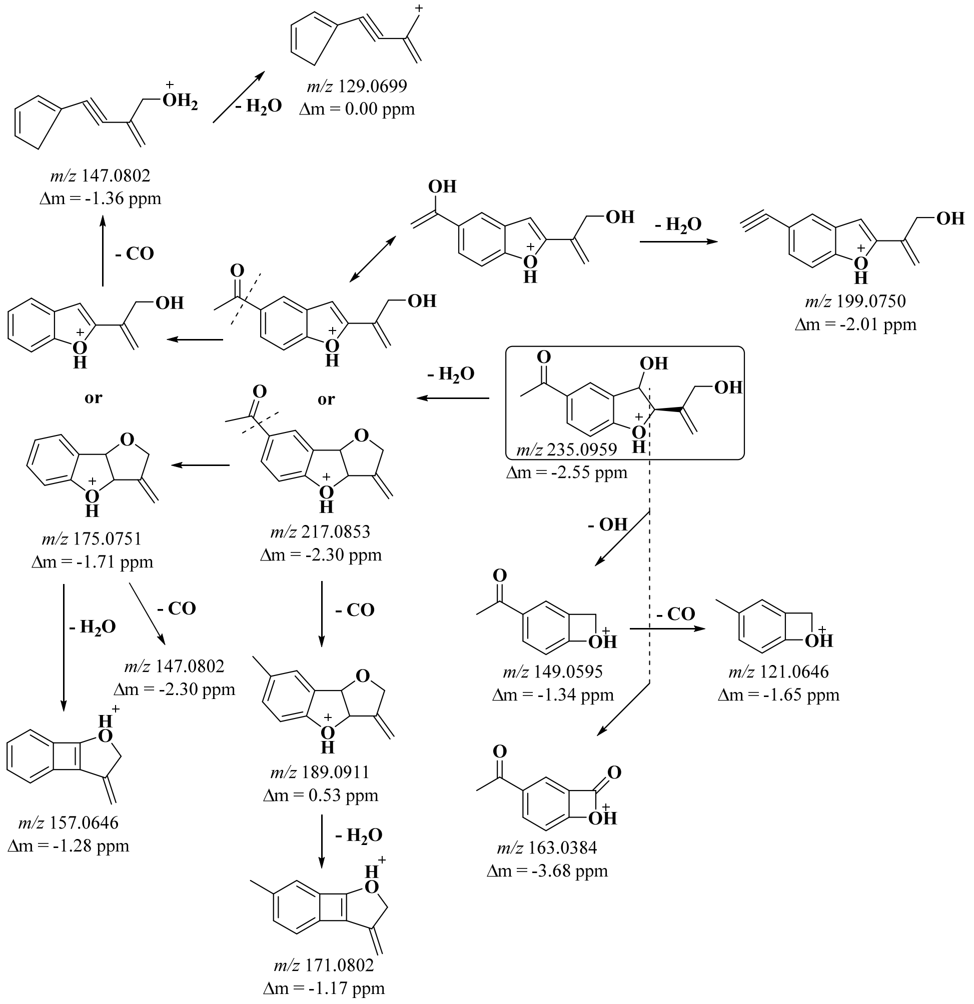

Figure S14. Proposed fragmentation of protonated gnaphaliol $[M+H]^+$.

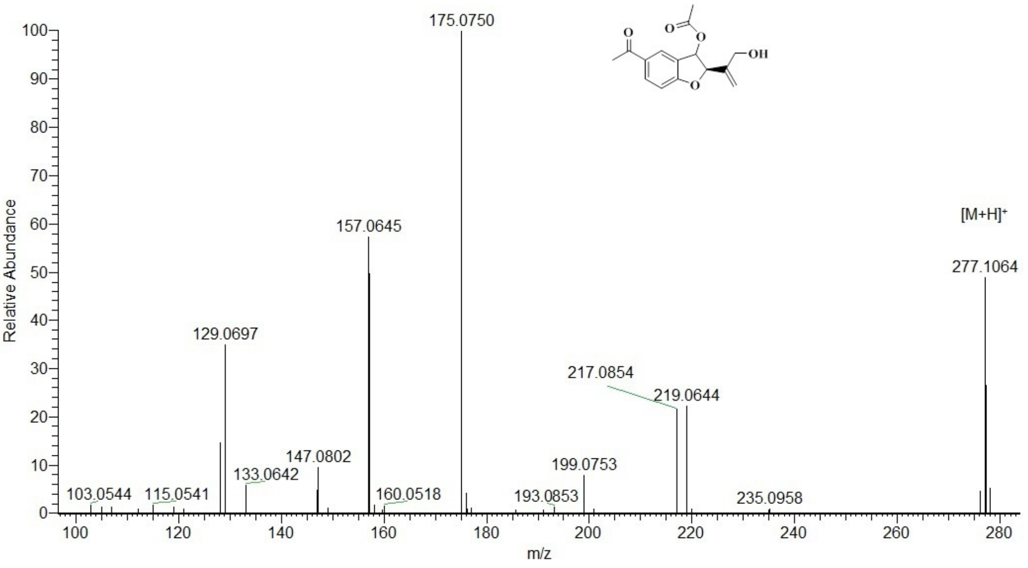

Figure S15. Mass spectrum of 3-actoxy-10-hydroxytremeton obtained by positive ion ESI-MS/MS

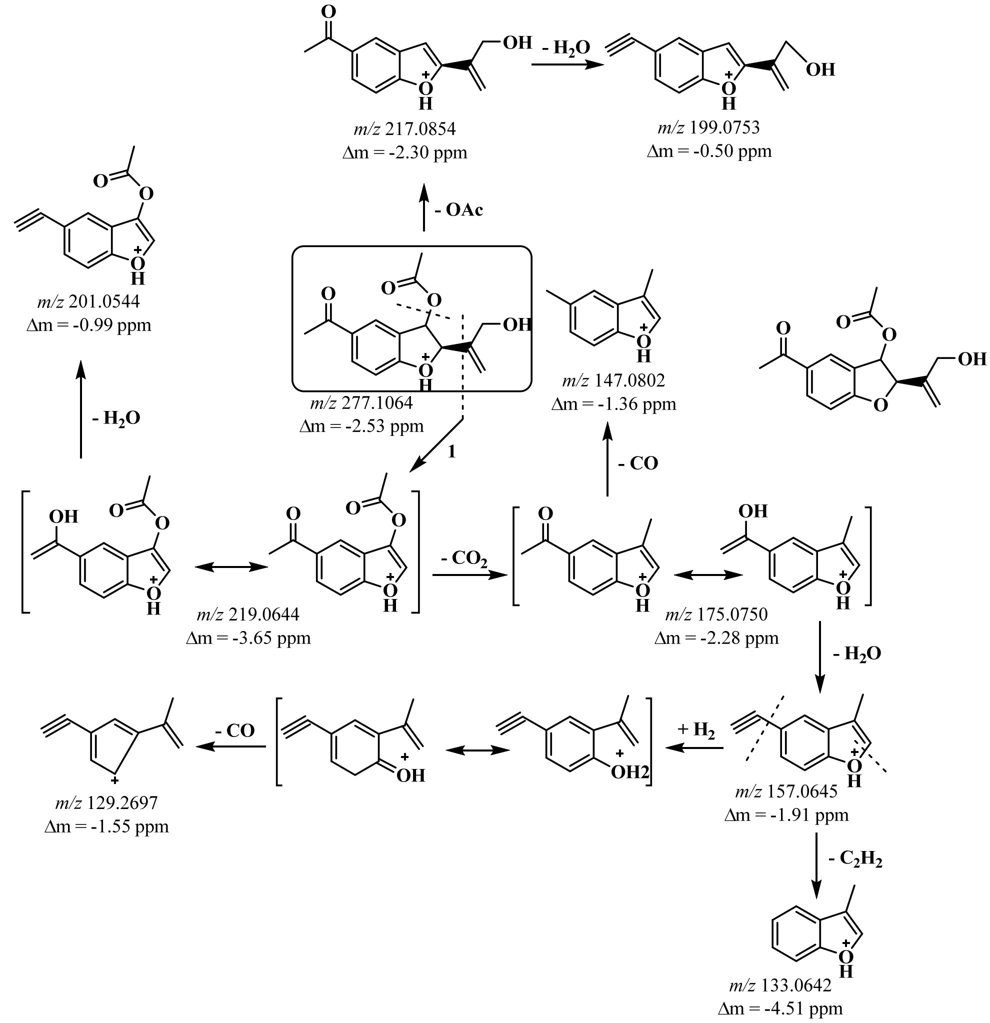

Figure S16. Proposed fragmentation of protonated 3-actoxy-10-hydroxytremeton $[M+H]^+$.

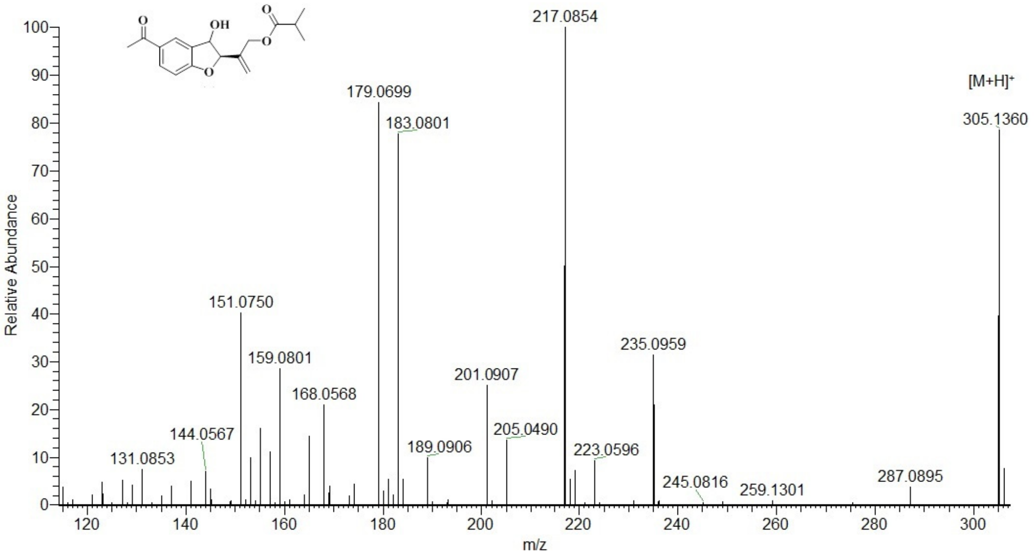

Figure S17. Mass spectrum of 13-(2-methylpropanoyloxy)toxol obtained by positive ion ESI-MS/MS

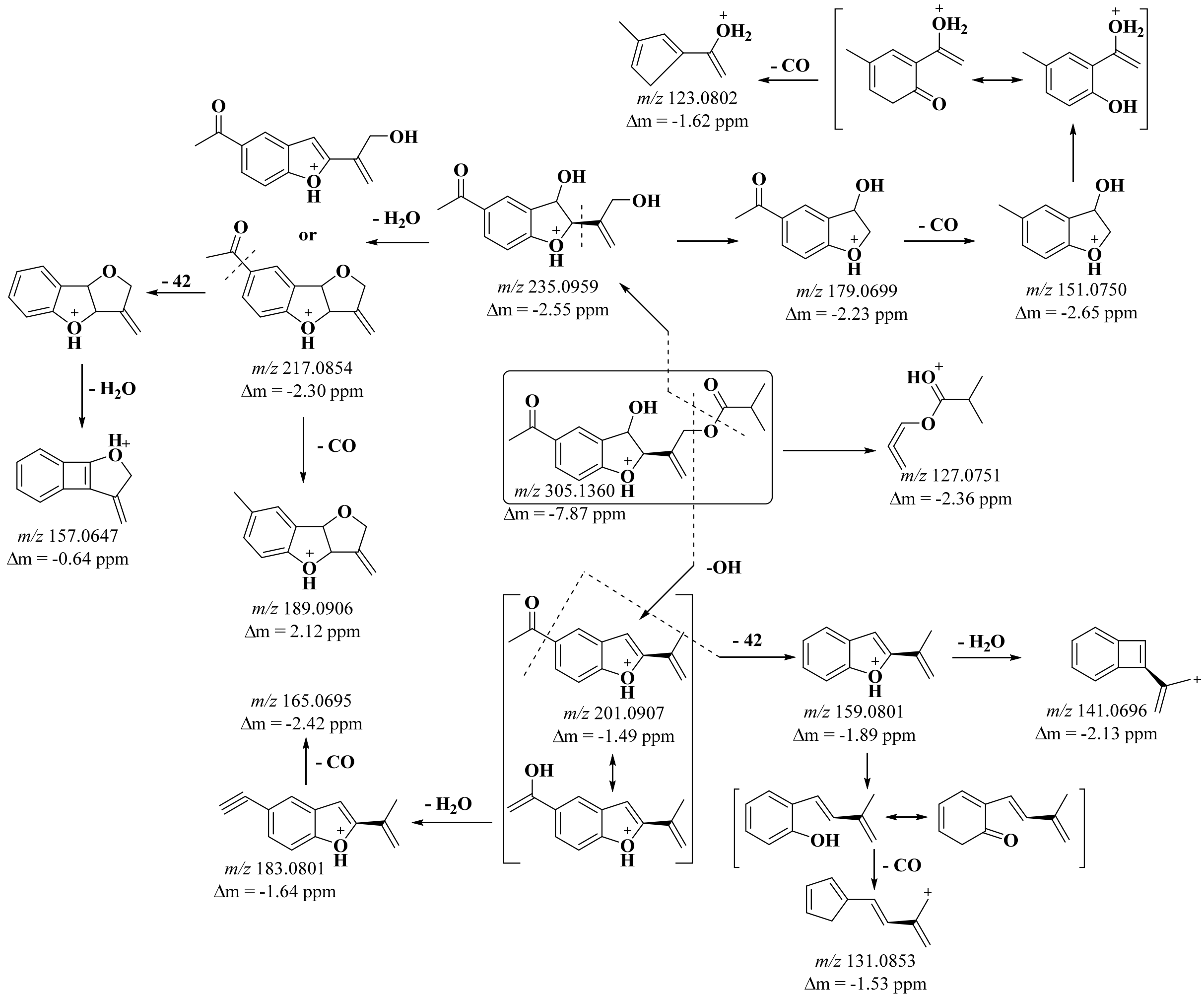

Figure S18. Proposed fragmentation of protonated 13-(2-methylpropanoyloxy)toxol $[M+H]^+$.

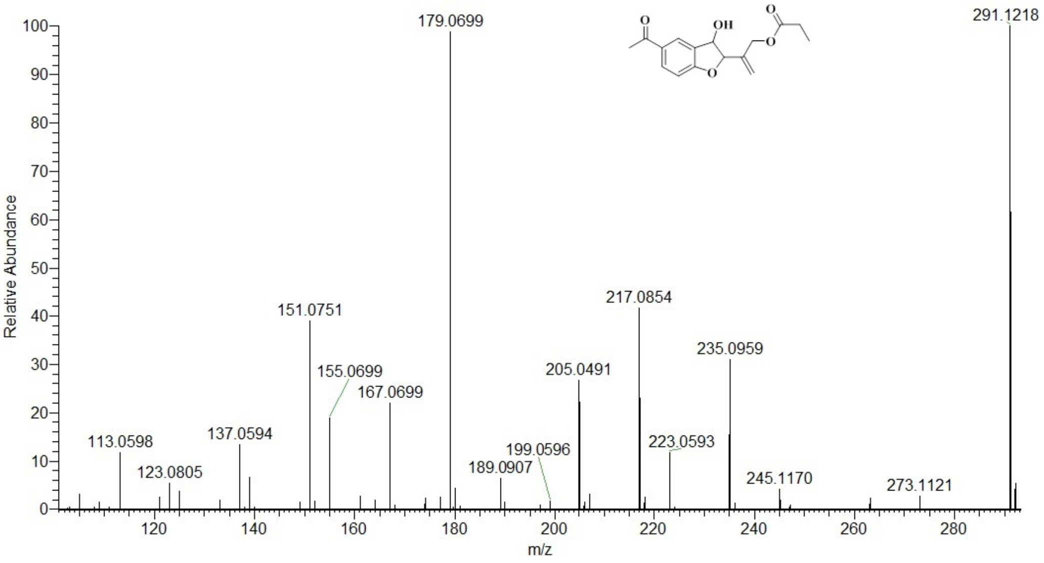

Figure S19. Mass spectrum of 3-hydroxy-10-propanoyloxy tremeton obtained by positive ion ESI-MS/MS

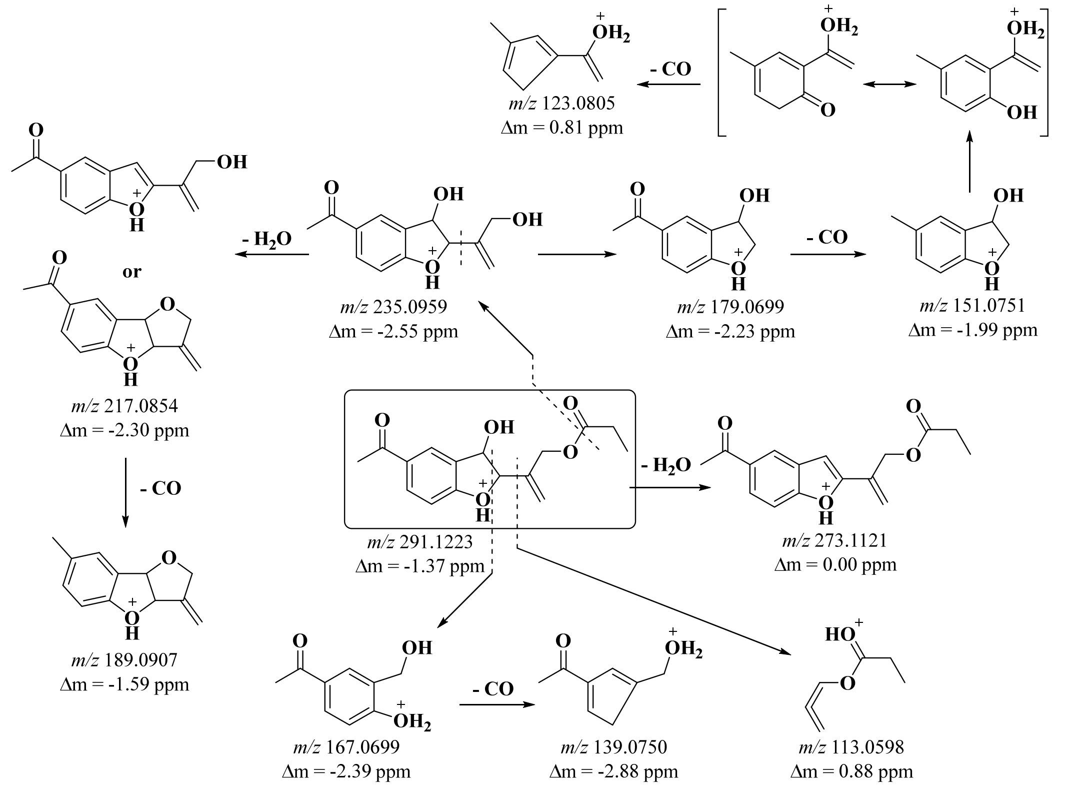

Figure S20. Proposed fragmentation of protonated 3-hydroxy-10-propanoyloxy tremeton $[M+H]^+$.

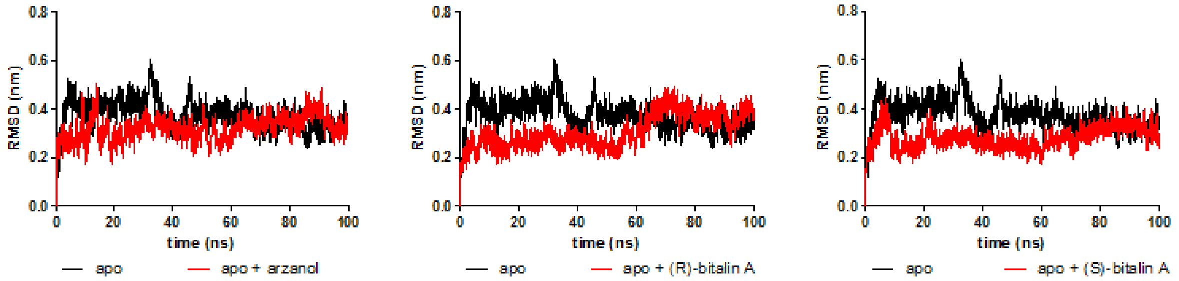

Figure S21. RMSD of protein backbone when complexed with arzanol, (R)-bitalin A and (S)-bitalin A compared with the and apo form

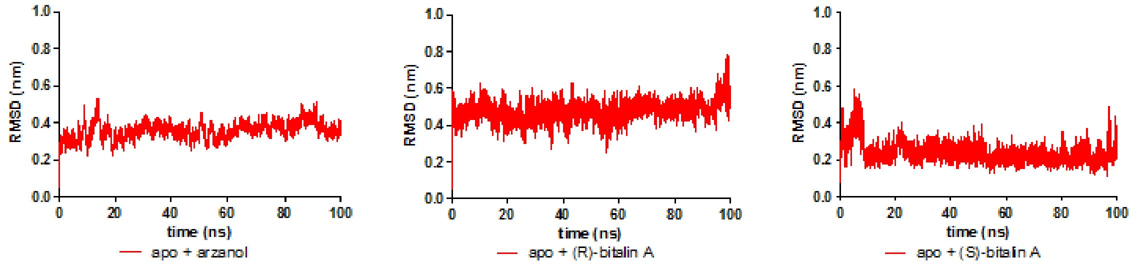

Figure S22. RMSD of arzanol, (R)-bitalin A and (S)-bitalin A when complexed with albumin

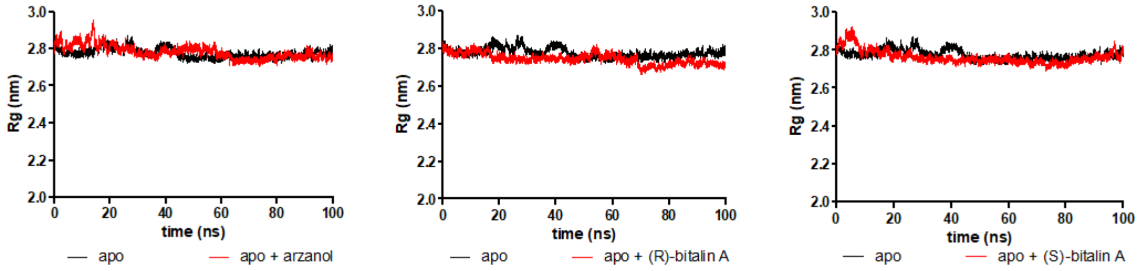

Figure S23. Radius of gyration of protein atoms when complexed with arzanol, (R)-bitalin A and (S)-bitalin A compared with the and apo form

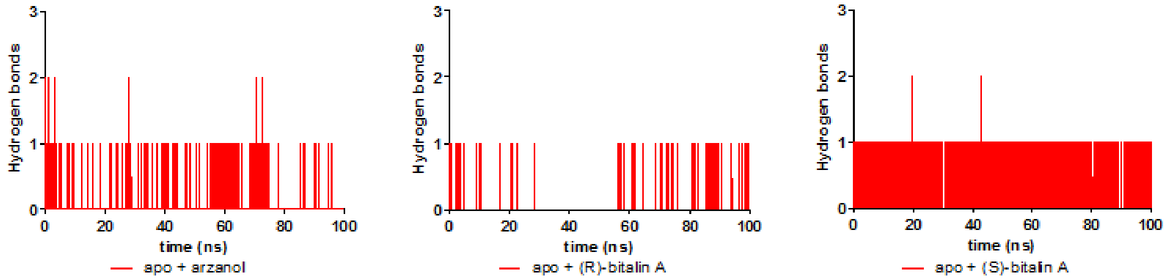

Figure S24. Number of hydrogen bonds between albumin and arzanol, (R)-bitalin A and (S)-bitalin A
